# Paired Pulsed Decarboxylative Hydroxylation Designed by Online Electrochemistry–Mass Spectrometry

**DOI:** 10.1021/jacs.5c12895

**Published:** 2025-10-02

**Authors:** Adarsh Koovakattil Surendran, Jana Roithová

**Affiliations:** Department of Spectroscopy and Catalysis, Institute for Molecules and Materials, 6029 Radboud University, Heyendaalseweg 135, 6525 AJ Nijmegen, The Netherlands

## Abstract

We present a novel strategy for optimizing paired pulsed electrosynthesis by real-time monitoring of redox processes at the electrode interface using voltammetry–electrospray ionization mass spectrometry (VESI-MS). VESI-MS provides molecular insight into interfacial events, enabling the rational design of voltage pulses and synchronization of redox reactions, facilitating the discovery of new alternating current (AC) electrochemical transformations. We apply this approach to develop a selective decarboxylative hydroxylation by coupling oxygen reduction with the oxidative decarboxylation of a carboxylic acid. The pilot study with 2-phenylpropionic acid yields 1-phenylethanol with excellent selectivity using only molecular oxygen, KPF_6_, water, and carbon electrodes. This work opens new possibilities for the design of pulsed electrosynthetic reactions.

## Graphical abstract

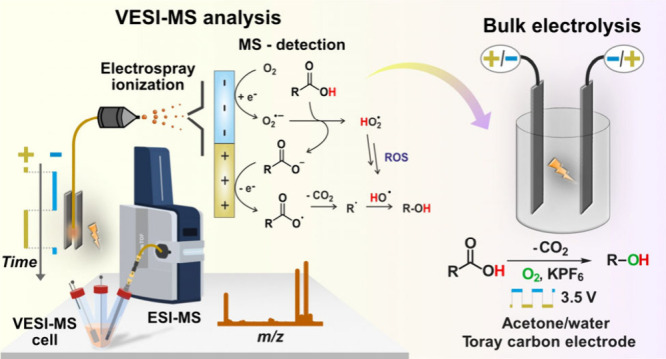

Electrosynthesis utilizes single or sequences of one-electron oxidation and reduction steps to enable clean chemical transformations that would typically require stoichiometric reagents, rare metal catalysts, or other activation methods.^1–8^ However, this seemingly ideal approach is often complicated by undesired effects associated with the electrosynthesis process. For example, mass transport effects can lead to the accumulation of intermediates, triggering unwanted side reactions, and causing issues such as overoxidation, over-reduction, or electrode fouling due to uncontrolled interfacial conditions.^9–12^ In many instances, effective chemical synthesis occurs at one electrode, while it is coupled with a sacrificial redox reaction at the other electrode (Figure 1a).^13,14^

**Figure 1:**
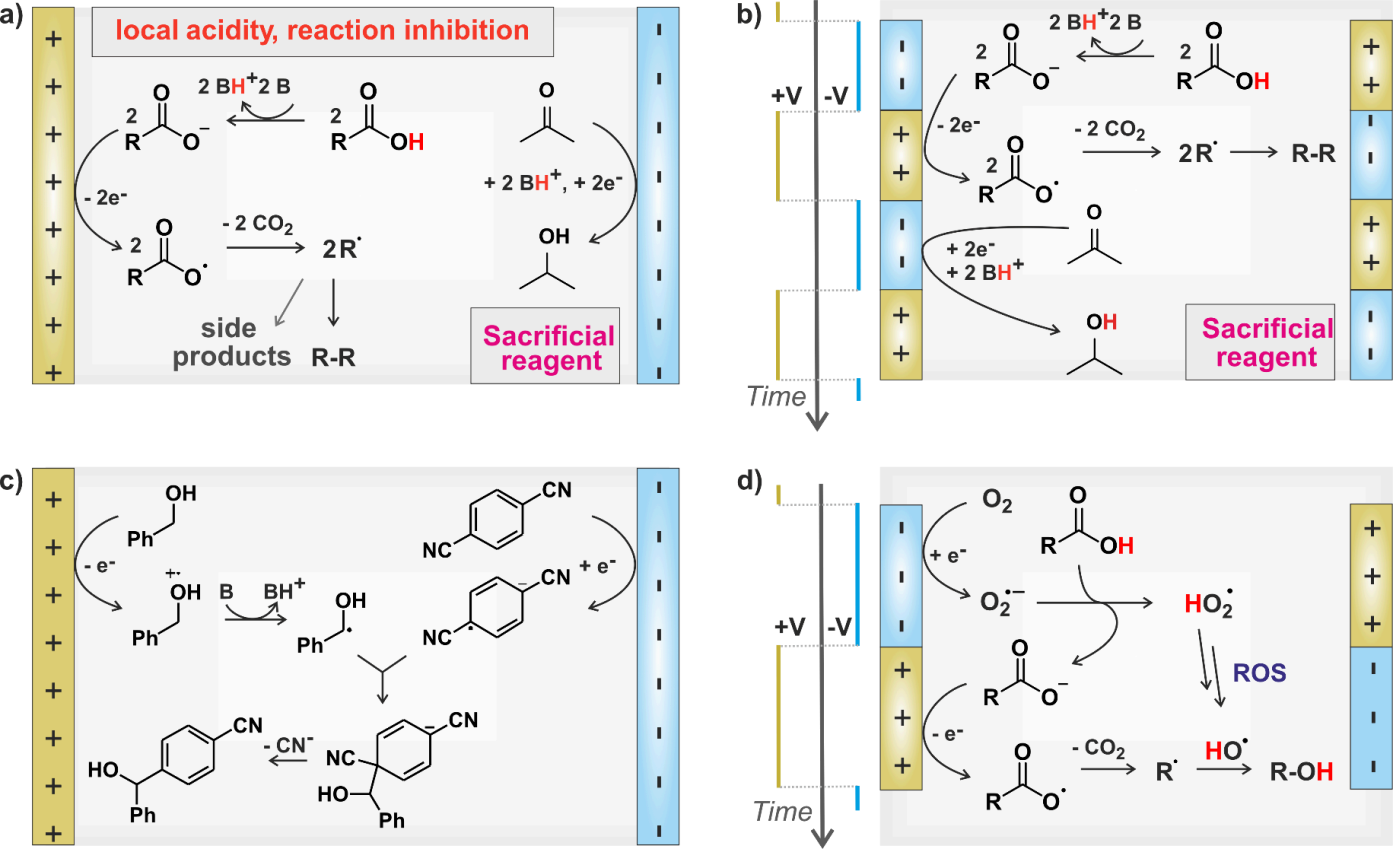
Examples of approaches to electrosynthesis. (a) DC Kolbe synthesis.^14,36^ (b) rAP Kolbe synthesis.^14^ (c) Convergent Paired Electrosynthesis.^22^ (d) Paired Pulsed Electrosynthesis (developed here).

Recent advancements in the field have demonstrated that utilizing alternating current or alternating voltage pulses (AVP) can help reduce mass transport effects, local pH variation, and minimize electrode fouling (Figure 1b).^14–18^ However, designing these pulses necessitates careful consideration of the chemistry at the electrode interface, which presents challenges that may hinder the development of AVP for electrosynthesis.^19–21^

Another promising development is the convergent pairing of electrochemical reactions, where both electrodes generate reactive intermediates that eventually couple to form a product (Figure 1c).^22–25^ The generality of this approach is often hindered by the spatial separation of the initially formed reactive species, which frequently requires the formation of persistent radicals or other stabilized intermediates.

Electrochemistry – mass spectrometry couplings can provide molecular insight into electrochemical reactions.^26–31^ We have recently developed a Voltammetry–Electrospray Ionization Mass Spectrometry (VESI-MS) approach to study chemical processes occurring near electrodes in real-time during the electrochemical reaction (Figures S1 and S2).^10,32^ In this paper, we demonstrate how this approach can be employed to tune voltage pulses and design pulsed paired electrosynthesis. We specifically illustrate its application in the decarboxylative hydroxylation reaction (Figure 1d). By utilizing alternating voltage pulsing, we can selectively produce the alcohol while suppressing both radical dimerization and overoxidation.^33–36^

The hypothesis of the reaction posits that electrochemical oxygen reduction (ORR) and carboxylic acid oxidation (CAO) are complementary redox reactions. ORR is favored in acidic conditions, whereas CAO requires a basic environment.^14,36–38^ The superoxide or peroxide species generated during the ORR can function as in situ bases, deprotonating carboxylic acids to produce carboxylates.^39,40^ These carboxylates are then oxidized during the subsequent anodic pulse, leading to decarboxylation and the formation of alkyl radicals.^11,36,41^ In the next step, the cathodic pulse reduces oxygen to hydroxyl radical or other reactive oxygen species (ROS), which may react with the alkyl radicals to form the corresponding alcohol.^42–44^ If both reactions are conducted using optimized alternating voltage pulses, the overall reaction would be

$$2\mathrm{RCOOH}+O_{2}\to 2R\mathrm{-OH}+2{\mathrm{CO}}_{2}$$

As oxidation and reduction alternate at each electrode, the same redox sequence occurs at both electrodes in tandem, eliminating the need for a sacrificial counter-reaction and enabling efficient paired electrolysis.

We tested our hypothesis with the VESI-MS experiments. First, we evaluated whether carboxylate can be generated during oxygen reduction on a Toray carbon electrode. We selected 2-phenylpropionic acid (RCOOH in the following) as the model substrate due to the stability of its secondary benzylic radical and cation. A linear sweep VESI-MS voltammogram from −0.2 to −1.95 V vs Pt, of a 2 mM RCOOH in acetone under O_2_ (0.12 bar) with KPF_6_ (2 mM) electrolyte confirms that carboxylate can be generated in situ during ORR (Figure 2a, see Figure S4 for the related mass spectra). The current caused by the oxygen reduction at the electrode correlates with the depletion of the carboxylic acid signal ([RCOOH]­K^+^) and a simultaneous rise of the signal of carboxylate anions ([RCOO^–^]­2K^+^). We use KPF_6_ rather than the commonly used tetrabutylammonium salts because it is cheaper and more compatible with ESI-MS measurements. In addition, potassium ions often bind to neutral molecules during the ionization, enabling their detection by ESI-MS, as observed here.

**Figure 2:**
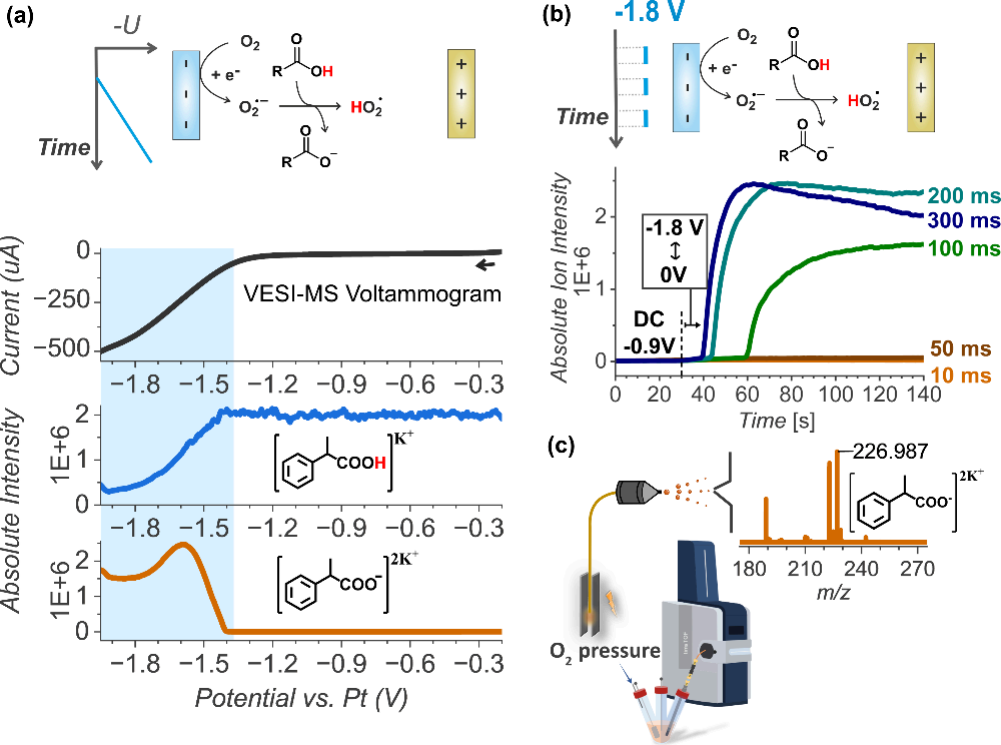
(a) Proof of concept: oxygen reduction at the electrode leads to carboxylic acid deprotonation. (b) Tuning the reduction pulse length for the maximum carboxylate formation. (c) VESI-MS setup.

To effectively couple the oxygen reduction reaction (ORR) with carboxylate oxidation, we need to estimate the time scales of each reaction: the duration of the reduction pulse for maximizing carboxylate formation and the optimal oxidation pulse length for complete decarboxylation. We focused first on optimizing the reduction pulse. We applied symmetric square wave pulses between −1.8 V (vs Pt) (where ORR and carboxylate formation occur) and 0 V (where no reaction takes place). Pulse durations were varied from 100 μs to 300 ms (100 μs, 1, 10, 50, 100, 200, and 300 ms), and the carboxylate formation was monitored in real-time with mass spectrometry. To establish a nonpulsed baseline, the electrode was held at −0.9 V (vs Pt) for 30 s, followed by pulsing for about 2 min to ensure adequate detection time. The transfer time between the electrode and ESI-MS detection was about 10 s. No carboxylate signal was observed for pulse durations below 10 ms, while a slight increase was seen at 10 ms, followed by a sharp rise at 100 ms, reaching a maximum at 200–300 ms (Figure 2b and Figure S5). The onset of the carboxylate signal is influenced by its concentration; lower concentrations require longer times for detectable accumulation. Hence, a 300 ms pulse maximizes the concentration of carboxylate, but we reach the diffusion limit since the same amount of carboxylate can be produced with 200 ms pulses over a longer period.

We chose to fix the 200 ms reduction pulse duration at −1.8 V and proceeded with optimizing the oxidation pulse. The oxidation voltage was set to 1.7 V (vs Pt) as determined from VESI-MS experiments with a constant −1.8 V (vs Pt) (200 ms) reduction pulse and systematically increasing the oxidation pulse potential from 1 to 1.7 V (vs Pt) (Figure S6, the pulse duration was symmetrical, 200 ms). At 1.7 V (vs Pt), all the carboxylate ions generated from the reduction pulse are oxidized, leading to the disappearance of the [RCOO^–^]­2K^+^ ions from the mass spectra. Next, we varied the oxidation pulse duration, keeping the offset of 1.7 V (vs Pt), from 100 μs to 600 ms, while monitoring the depletion of the carboxylate signal (Figure 3a and Figure S7, the reduction pulses were kept constant at −1.8 V (vs Pt) for 200 ms). The experimental sequence again started by holding the average DC potential (−0.05 V) for 30 s, followed by recording the effects of pulsing for 2 min.

**Figure 3:**
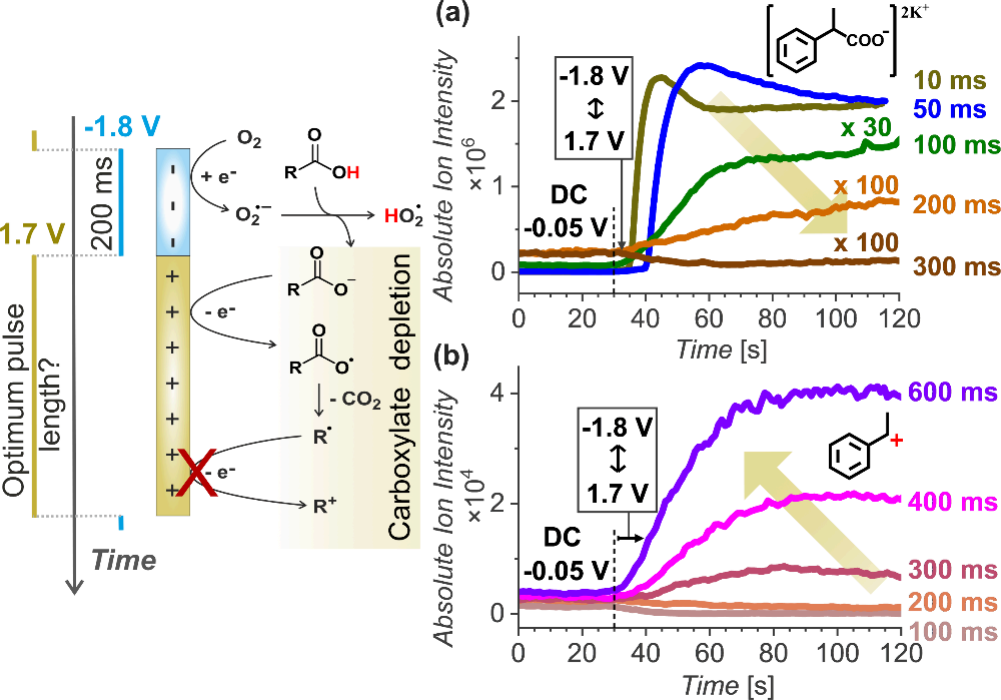
Pairing the 200 ms reduction pulses with the oxidation pulses.

At short oxidation pulse durations (below 50 ms), the carboxylate formed during the previous 200 ms reduction pulse remains largely unoxidized, and the [RCOO^–^]­2K^+^ signal remains unchanged (Figure 3a). With longer oxidation pulses, the signal gradually decreases, indicating the consumption of carboxylates. When using a 200 ms oxidation pulse, nearly all the carboxylate is oxidized. With a 300 ms pulse, the carboxylate signal drops below the baseline, indicating the complete oxidation of the carboxylate produced during the reduction pulse and the further consumption of carboxylate accumulated in the solution from earlier measurements. Further prolongation of the oxidation pulses, to 300 ms and longer, resulted in observation of the corresponding carbocations, indicating undesired overoxidation (Figure 3b and Figure S8).

The results indicate that symmetrical pulses of oxidation and reduction yield the optimal conditions, which is also the ideal scenario for pulsed pairing because the processes at both electrodes are symmetrical. With these molecular insights and the optimal pulse durations, we performed bulk electrolysis using the rapid alternating polarity (rAP) function of the ElectraSyn, starting with 200 ms pulses at a cell voltage of 3.5 V, utilizing identical Toray carbon electrodes (2 cm^2^) for both the anode and cathode (Figure 4). In an 8 mL solution of 2-phenylpropionic acid (0.125 M) and KPF_6_ (0.1 M) dissolved in acetone, we added 1 M of water to enhance the solubility of the carboxylate. Bulk electrolysis lasted 5 h with continuous O_2_ bubbling (35 mbar) and stirring. Afterward, we evaporated the acetone and dissolved the residue in CDCl_3_, adding trimethoxybenzene as an NMR internal standard. The insolubility of KPF_6_ in chloroform allowed for easy separation from the reaction mixture. ^1^H NMR analysis confirmed the formation of 1-phenylethanol with a yield of 12.1% over 5 h (Figure S9).

**Figure 4:**
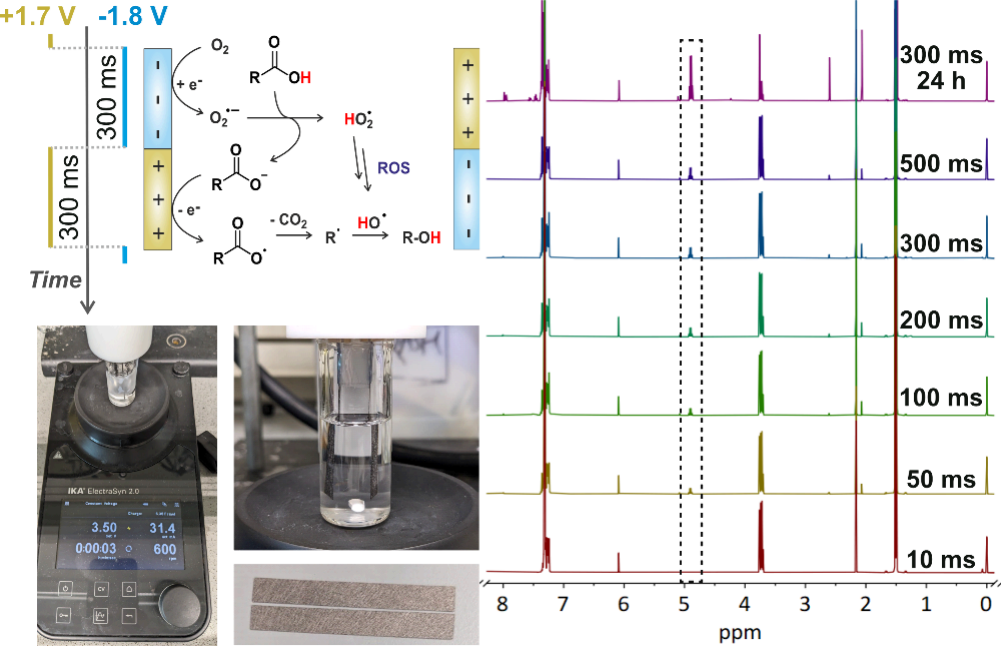
Paired Pulsed Electrosynthesis. Decarboxylative hydroxylation was performed in a 2-electrode cell at a cell voltage of 3.5 V using carbon-paper electrodes.

To determine if the optimal conditions identified by VESI-MS apply at a preparative scale, we conducted additional bulk electrolysis with pulse durations from 10 to 500 ms (see Table 1). The alcohol yield increased with the pulse duration, and the charge consumption decreased from 11.6 F/mol at 10 ms to 6.3 F/mol at 300 ms (Table S1). Alternating current (AC) electrolysis requires more charge than DC electrolysis due to the continuous restructuring of the double layer during polarity switching. Hence, higher AC frequencies result in higher energy losses. Here, longer pulses enhance reaction yield while reducing energy losses from non-Faradaic charging.

**Table 1:** Pulsed Paired Decarboxylative Hydroxylation of 2-Phenylpropionic Acid^a^

pulse length [ms]	1-phenyl ethanol [%]	acetophenone [%]	alcohol selectivity [%]
10	1.1	Traces	99.9
50	7.8	0.13	98.4
100	8.9	0.15	98.3
200	12.1	0.24	98.0
300	13.9	0.29	97.9
500	16.2	0.43	97.4
300^b^	61.2	4.40	93.3
300^c^	8.4	not formed	100
300^d^	9.1	0.95	90.5
DC	0.8	4.59	15.3^e^

a Standard conditions: 2-phenylpropionic acid (125 mM) with KPF_6_ electrolyte (100 mM) in 1 M water in acetone (8 mL) for 5 h with continuous bubbling O_2_ at 35 mbar.

b Standard conditions, but the reaction time was 24 h.

c Standard conditions, but without oxygen (under argon); instead of O_2_ reduction, acetone to isopropanol reduction was observed (Figures S14 and S15).

d Standard conditions, without water (Figure S16).

e Other aromatic products were formed too. The selectivity refers only to the ratio of alcohol to ketone.

Finally, we extended the electrolysis for 24 h using 300 ms pulses, yielding 61.2% of the alcohol with only 4.3% acetophenone and no signs of electrode fouling, making the process well-suited for long-term operations (Figure S10 and S11). To confirm that alternating polarity is essential for the reaction, we performed a control DC electrolysis at 3.5 V for 5 h under otherwise identical conditions. Only a trace amount of 1-phenylethanol was observed (0.8%), along with a complex mixture of unidentified byproducts (Figure S12). In contrast to the clean electrode surface after AC electrolysis, noticeable deposition was observed on the electrode after just 5 h under DC conditions (Figure S13).

To test if water plays a role beyond solubilizing the carboxylate, AC electrolysis with 300 ms pulsing under argon with 1 M water was performed. The yield dropped from 13.9% to 8.4%. The substantial product formation suggests an alternative pathway for generating local O_2_ and ROS, likely via water oxidation. The counter-reaction is the acetone reduction to isopropanol and related products, which were detected only in the absence of added O_2_ (Table S1, Figure S14 and S15).^45–48^ AC electrolysis under O_2_ at 300 ms without added water yielded 9.1% (vs 13.9% with both O_2_ and water) (Table S1 and Figure S16). The improved carboxylate solubility in the presence of water, combined with synergistic generation of ROS from both O_2_ and H_2_O, most likely accounts for the higher yield when both are present.

We also tested the VESI-MS analysis for a nonbenzylic substrate, 4-phenylbutyric acid (Figures S17–S25). A longer pulse (300 ms) was required to maximize carboxylate formation (Figure S19); all other parameters remained the same. Without any optimization, the bulk electrolysis for 5 h at 300 ms symmetrical pulses yields a mixture of alcohols: 3-phenyl-1-propanol, 1-phenyl-2-propanol, and 1-phenyl-1-propanol with a combined yield of 2.8% (Figures S26 and S27).

In summary, we report a new approach to designing pulsed paired electrosynthesis. We demonstrate the approach for the first electrosynthetic selective route to decarboxylative hydroxylation, achieved by coupling carboxylic acid oxidation with dioxygen reduction. This reaction proceeds under exceptionally simple and sustainable conditions, using only a carboxylic acid, molecular oxygen, water, and KPF_6_ in acetone on inexpensive carbon electrodes, with no added reagents, catalysts, or sacrificial agents.^49^ Central to this discovery is our integrated VESI-MS platform, which enables real-time monitoring of interfacial speciation and was crucial for the rapid testing of reaction hypotheses and identifying the reaction time scale required to access the desired reactivity. The method yields up to 60% with excellent selectivity and long-term electrode stability. Ongoing efforts are focused on expanding the substrate scope and further refining the reaction conditions, including testing under flow electrochemical conditions. This work not only establishes a new green transformation but also highlights the potential of VESI-MS to unlock electrosynthetic processes that would otherwise be inaccessible due to the complexity of alternating current systems.

## Supplementary Material



## Notes

Experimental details, ESI-MS mass spectra, and additional experiments (PDF)

## Abbreviations

ORR: Oxygen Reduction Reaction
ESI-MS: Electrospray Ionization Mass Spectrometry
VESI-MS: Voltammetry – Electrospray Ionization Mass Spectrometry
