# Supplementary material for: Paired Pulsed Decarboxylative Hydroxylation Designed by Online Electrochemistry–Mass Spectrometry
Source: J Am Chem Soc. 2025 Oct 2;147(41):36975–9. doi: 10.1021/jacs.5c12895 (PMC12532190; doi:10.1021/jacs.5c12895)
Supplement: Supplementary file 1 [file ja5c12895_si_001.pdf]

Supplementary Information for

Paired Pulsed Decarboxylative Hydroxylation Designed by Online Electrochemistry–Mass Spectrometry

Adarsh Koovakattil Surendran and Jana Roithová*

Department of Spectroscopy and Catalysis, Institute for Molecules and Materials, Radboud University, Heyendaalseweg 135, 6525 AJ Nijmegen, The Netherlands.

Corresponding author: j. roithova@science.ru.nl

Table of Contents

1. Materials and Methods

1.1. General Information....S3

1.2. VESI-MS Analysis....S3

1.3. General procedure for the bulk electrolysis S7

2. Experimental Results for 2-phenylpropionic acid

2.1. Mass spectra recorded during VESI-MS scan....S8

2.2. Mass spectra recorded during optimization of the reduction pulses....S9

2.3. Estimation of the oxidation potential....S10

2.4. Mass spectra recorded during optimization of the oxidation pulses....S11

2.5. ^{1}H NMR after 5 h bulk electrolysis with 200 ms pulses....S13

2.6. ^{1}H NMR after 24 h bulk electrolysis with 300 ms pulses....S14

2.7. Electrodes after 24 h bulk electrolysis with 300 ms pulses....S15

2.8. ^{1}H NMR after 5 h bulk electrolysis under DC....S16

2.9. Electrodes after 5 h bulk electrolysis under DC....S17

2.10. ^{1}H NMR after 5 h AC (300 ms) electrolysis under argon with 1 M H_2O....S18

2.11. ^{1}H NMR after 5 h AC (300 ms) electrolysis under O_2 without H_2O....S21

2.12. Summary of bulk electrolysis....S22

3. Experimental Results for 4-phenylbutyric acid

3.1. VESI-MS monitoring – ion traces....S24

3.2. VESI-MS monitoring – mass spectra....S25

3.3. Optimization of the reduction pulse length – traces of carboxylate ion....S26

3.4. Optimization of the reduction pulses – mass spectra of carboxylate....S27

3.5. Estimation of the oxidation pulse potential....S28

3.6. Optimization of the oxidation pulse length – traces of carboxylate ion....S29

3.7. Optimization of the oxidation pulse length – traces of carbocation....S30

3.8. Optimization of the oxidation pulses – mass spectra of carboxylate....S31

3.9. Optimization of the oxidation pulses – mass spectra of carbocation....S32

3.10. ^{1}H NMR after 5 h bulk electrolysis with 300 ms pulses....S33

4. References

1. Materials and Methods

1.1. General Information

All chemicals were purchased from commercial sources and used without further purification. Potassium hexafluorophosphate (99%) and deuterated chloroform (99.8 atom% D) were obtained from Sigma-Aldrich, 4-phenylbutyric acid and analytical grade acetone from VWR Chemicals. (±)-2-phenylpropionic acid (98%) was sourced from Fluka, and 1,3,5-trimethoxybenzene from Fluorochem. Acetone-d_6 was from Deutero GmbH. Toray carbon paper (TGP-H-060) was obtained from Fuel Cell Earth. Platinum wire and platinum gauze (99.9%) were from Alfa Aesar. The fused silica capillary (ID 100 µm, OD 190 µm, part #: Z-FSS-100190) was purchased from Postnova.

A Metrohm potentiostat (PGSTAT204) was used for VESI-MS experiments. A digital electronic back pressure regulator (EL-PRESS P-702CV-21KR-RAD-11-K) obtained from Bronkhorst was used to monitor and maintain a constant headspace gas pressure. VESI-MS analysis was conducted with a Bruker trapped ion mobility time of flight (timsTOF) mass spectrometer with an electrospray ionization (ESI) source. In this study, the timsTOF was operated as a typical TOF mass spectrometer with the ion mobility separation turned off. The following ESI settings were used to transfer the ions: capillary voltage 4 kV, dry heater 200 °C, dry gas 3 L/min, and nebulizer gas 0.5 bar. The detector (TOF) was mass-calibrated before the measurements using a low-concentration tuning mix (ESI-L, Part No. G1969-85000) from Agilent Technologies. Bulk electrolysis was performed using IKA ElectraSyn 2.0 setup with Toray carbon paper as the electrode material (visit IKA website for additional details https://www.ika.com/en/Products-LabEq/Electrochemistry-Kit-pg516/ElectraSyn-20-Package-20008980/).

1.2. VESI-MS Analysis

The VESI-MS setup consists of a single-compartment, gas-tight voltammetric cell equipped with a Pt pseudo-reference electrode, a Pt mesh counter electrode, and a custom-designed Toray carbon working electrode. Its configuration closely mirrors that of a conventional voltammetric cell. The key principle of the experiment lies in capturing and transferring species generated at or near the working electrode surface to the mass spectrometer. The most effective design employs a working electrode constructed from two sheets of Toray carbon paper, with a silica capillary sandwiched between them (see Figure S1 for the electrode assembly). This capillary extracts in-situ generated species directly from the electrode surface and delivers them to the mass spectrometer, driven by a gas-induced overpressure flow. The flow rate is regulated by adjusting the applied headspace gas pressure. A constant pressure maintained throughout the measurement via a digital electronic back pressure regulator (Bronkhorst, EL-PRESS P-702CV-21KR-RAD-11-K) connected to the cell's headspace. The VESI-MS cell polarization during experiments was controlled using a Metrohm potentiostat (PGSTAT204). Comprehensive details on the development, validation, and technical aspects of the VESI-MS method are available elsewhere.[1,2]

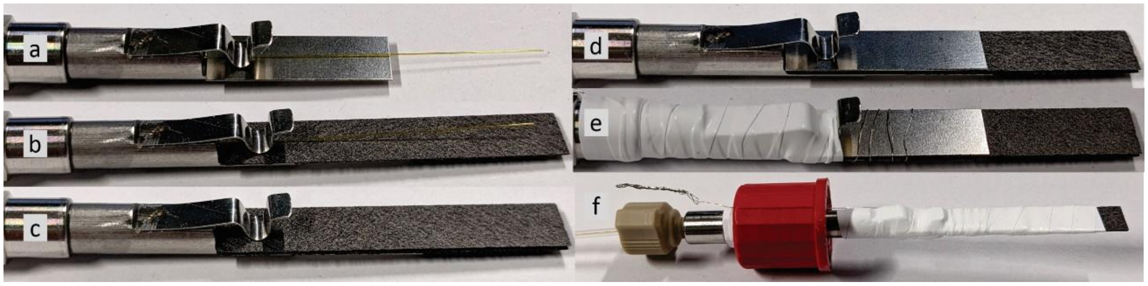

Figure S1: VESI-MS electrode assembly (reproduced from ref. 1). (a) A silica capillary is inserted through the electrode holder and on top of the stainless-steel (SS) support plate (also the contact plate; size: 0.7 cm x 3 cm). (b) Toray carbon paper (TCP, 0.7 cm x 4.3 cm) is inserted between the capillary and the back support plate, and the capillary is aligned in the middle of the carbon paper, with the end at approximately 0.2 to 0.3 cm from the bottom of the TCP. (c) The second TCP sheet is placed, sandwiching the capillary (care is taken to keep the capillary aligned in the middle). (d) The front SS support plate is inserted. (e) Contact wire is wound around the support plate, and the assembly is partially tightened by Teflon tape. (f) In the final stage, Teflon is wrapped until approximately 0.1 mm from the tip of the capillary, and the whole assembly is firmly tightened. The Teflon wrapping holds the assembly together, keeping the capillary in contact with the carbon paper. The capillary–electrode distance is set by the wall thickness of the capillary (50 µm, outer–inner radius). In the final setup, the capillary stays centered, with TCP sheet ends spaced by its outer diameter (190 µm). The carbon paper is rinsed with reaction solvent to ensure a clean surface.

The VESI-MS method allows us to monitor the formation of charged species at the electrode surface during a voltammetric scan, constant applied potential, or potential pulses. The VESI-MS voltammograms were recorded at a 5 mV s^{-1} scan rate in a solution of 1 M water in acetone, containing 2-phenylpropionic acid (2 mM), potassium hexafluorophosphate (KPF_6) electrolyte (2 mM), under a constant oxygen overpressure (0.12 bar). All VESI-MS experiments were conducted using a three-electrode configuration with a platinum wire serving as the pseudo-reference electrode. The cell was filled with 3 mL of solution, and it was used for multiple experiments until sufficient volume (~1.2 mL) remained to submerge the contact area of the electrode. For each 3 mL starting solution, multiple LSV scans (3 to 4) were first recorded at 5 mV s^{-1} over the range −0.2 V to −1.8 V, until the reference electrode potential was stabilized and the scans were reproducible. Once a stable LSV scan was obtained, the same solution was used to optimize the reduction pulses (10–400 ms). If the remaining volume was insufficient, the cell was flushed and refilled with a fresh solution. LSV scans were then repeated to confirm reproducibility with the prior measurement, followed by the remaining pulse experiments, including determination of the oxidation potential and optimization of the oxidation pulse duration. After each measurement, the electrode was held at a DC potential (–0.2 V, which is the starting potential for LSVs, or the respective DC hold used for pulse optimizations) to fast equilibrate the electrode potential for the next experiment. During this hold, the signal was monitored to allow transfer of any

residual carboxylate or carbocation species until a stable baseline was obtained. These minor residuals did not affect subsequent measurements, since a stable baseline was always established at the respective DC hold potential (~30 s) before applying pulses. The effect of the pulses was therefore quantified as the signal variation relative to this baseline.

The electrolyte's choice (KPF_6) and its concentration are based on their compatibility with mass spectrometry measurements. A higher concentration of the supporting electrolyte can cause ion suppression, electrospray discharge, and solidification, potentially blocking the entrance of the mass spectrometer. The transfer time for the species from the electrode surface to the mass spectrometer was 10 seconds.

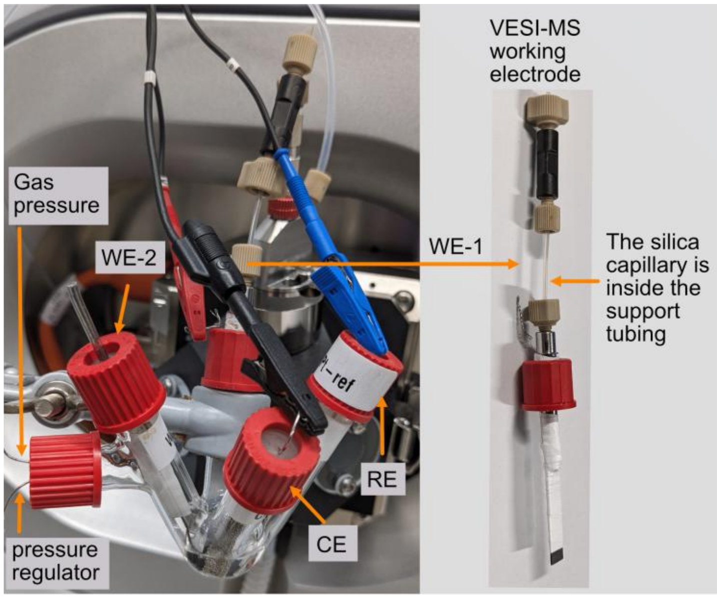

Figure S2: The VESI-MS cell coupled to the electrospray ionization (ESI) mass spectrometer (reproduced from ref. 2). WE-1: Primary VESI-MS working electrode is connected to the mass spectrometer through a silica capillary, RE: Platinum pseudo reference electrode, CE: platinum mesh counter electrode, WE-2: a low surface area secondary working electrode (from the same Toray carbon paper of the WE-1) is used to obtain the reference/trial voltammograms and the identified electrochemical potential window of interest is scanned on the primary VESI-MS working electrode at a slow scan rate 5 mV s^{-1}.

1.3. General procedure for the bulk electrolysis

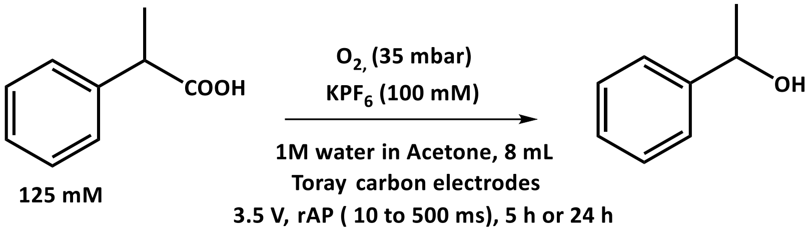

Preparative-scale electrolysis was carried out using the ElectraSyn 2.0 setup.[3] The ElectraSyn vial was charged with 8 mL of a solution containing 2-phenylpropionic acid or 4-phenylbutyric acid (125 mM), KPF_6 electrolyte (100 mM), and 1 M water in acetone. The cell was equipped with two identical Toray carbon electrodes, each with a wetted surface area of 2 cm^2, along with a stirring bar and a needle for oxygen bubbling. Oxygen was continuously bubbled through the reaction mixture at an overpressure of 35 mbar, and the solution was stirred at 600 rpm throughout the electrolysis.

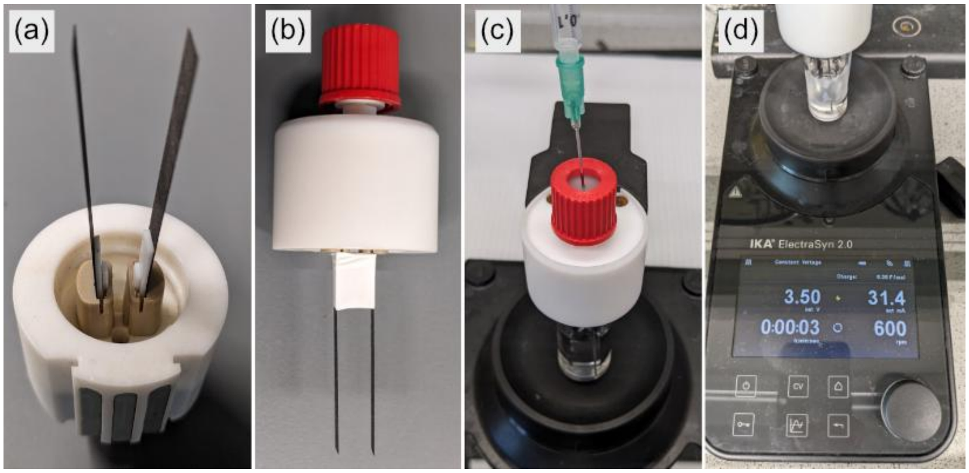

Figure S3: ElectraSyn 2.0 setup. (a) Toray carbon paper (0.7 cm x 6 cm) electrodes attached to the electrode holder (carbon paper electrode is not part of IKA ElectraSyn 2.0 kit, pieces of teflon inserts (0.5 mm thickness) were used to attach it to the holder). (b) Electrodes wrapped with teflon tape to ensure a parallel spacing. (c) Oxygen inlet needle inserted through the septum, maintaining the cell headspace at ~35 mbar O_2 pressure. A slight leakage ensures continuous bubbling of O_2 into the solution at this pressure. (d) ElectraSyn 2.0 under operation.

Electrolysis was performed using a two-electrode configuration without a reference electrode, applying a cell voltage of 3.5 V under rapid alternating polarity (rAP) conditions for 5 h or 24 h (extended electrolysis). A range of rAP switching intervals was tested, from 10 ms to 500 ms. After electrolysis, the electrodes were rinsed with acetone, and the solvent was removed under vacuum. To the residue, 4 mL of $CDCl_3$ was added along with 100 μL of 1,3,5-Trimethoxybenzene (250 mM) as an internal standard. The mixture was sonicated for 1 minute, and the insoluble KPF_6 was removed by syringe filtration. The resulting $CDCl_3$ solution was used for NMR analysis. The NMR yield was calculated using the aromatic-H (3H, s) signal of the internal standard at 6.09 ppm and the benzylic-H of the product 1-phenylethanol (1H, q, 4.93-4.88 ppm).

2. Experimental Results for 2-phenyl propionic acid

2.1. Mass spectra during VESI-MS scan

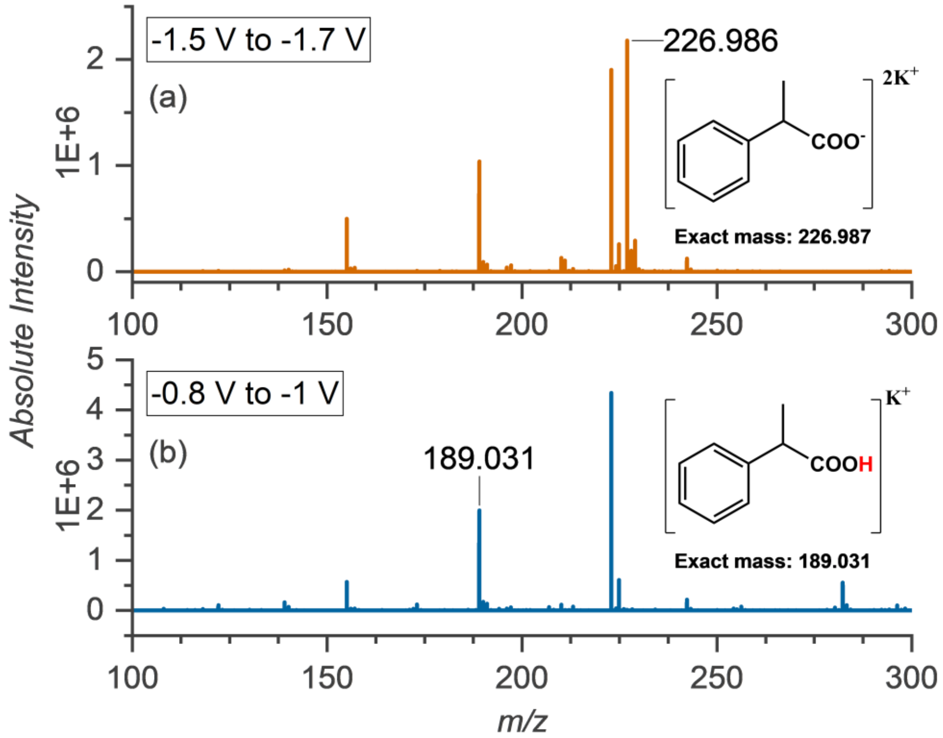

Figure S4: Mass spectra of 2-phenylpropionic acid and its carboxylate ion (m/z 226.987) recorded during the VESI-MS scan toward the oxygen reduction reaction, averaged over the indicated voltage ranges (vs. Pt). Experimental conditions: 2 mM 2-phenylpropionic acid, 2 mM KPF_6 in 1 M water in acetone, under 0.12 bar O_2 overpressure.

2.2. Mass spectra recorded during optimization of the reduction pulses

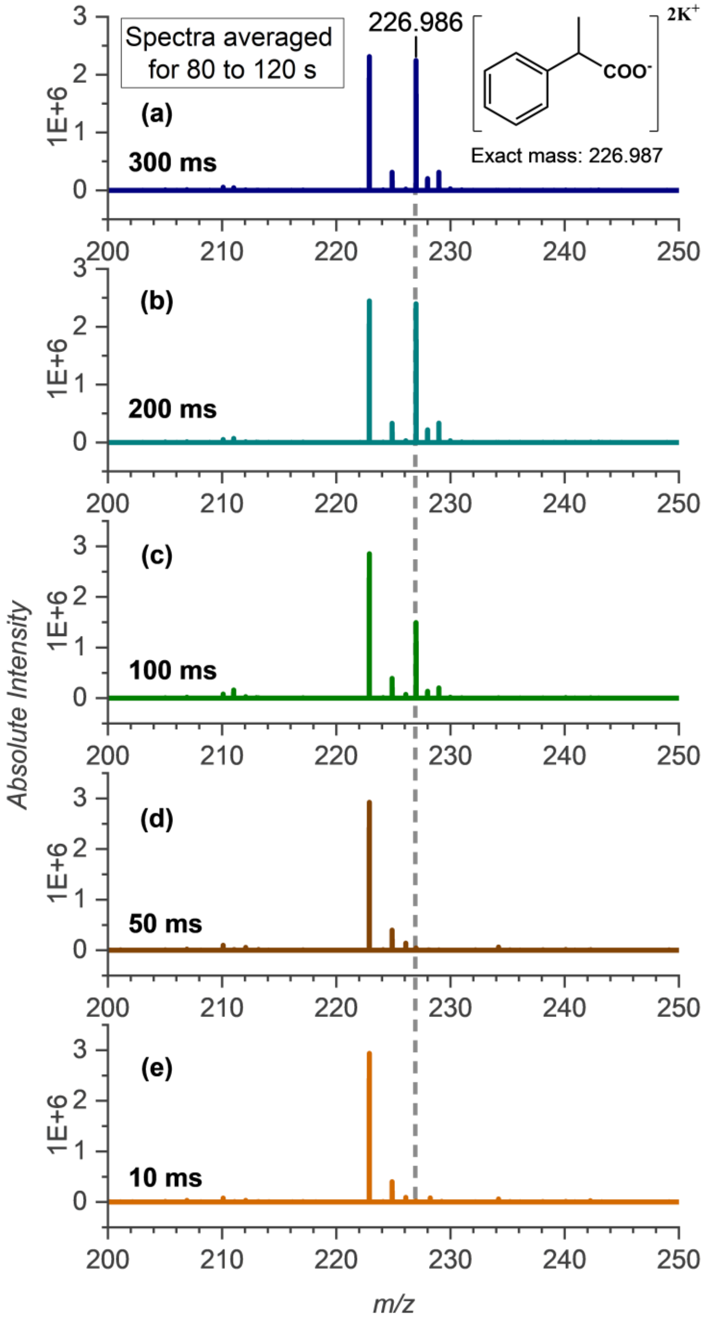

Figure S5: Mass spectra of 2-phenylpropionate ($[RCOO^{-}]^{2K^{+}}$) (m/z 226.987) detected during pulsing between -1.8 V (ORR) and 0 V (no reaction), with varying pulse lengths. Spectra were averaged over 80 - 120 s for: (a) 300 ms, (b) 200 ms, (c) 100 ms, (d) 50 ms, and (e) 10 ms. Experimental conditions: 2 mM 2-phenylpropionic acid, 2 mM KPF_6 in 1 M water in acetone, under 0.12 bar O_2 overpressure.

2.3. Estimation of the oxidation potential

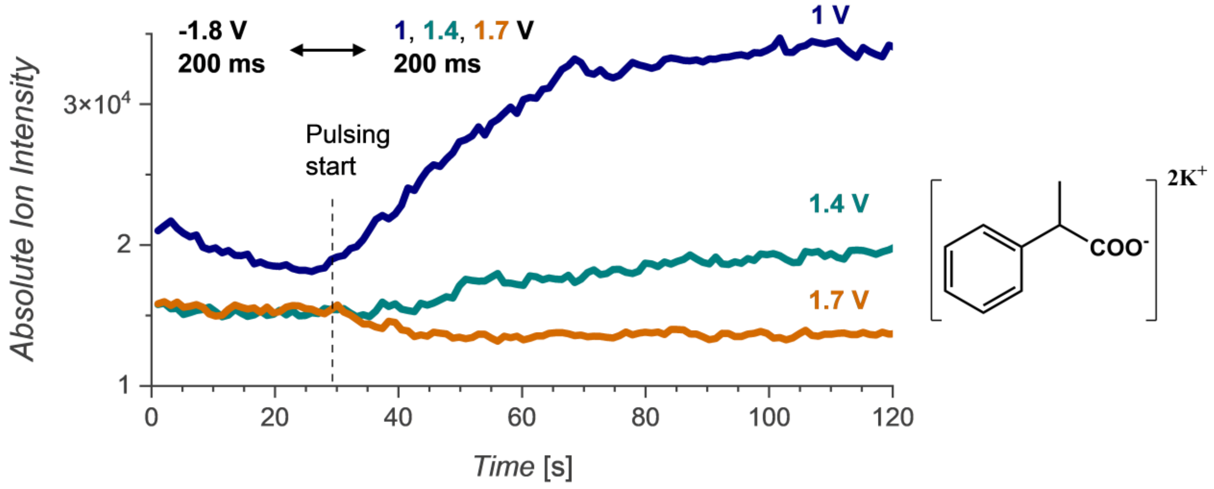

Figure S6: Extracted ion traces of 2-phenylpropionate ($[RCOO^-]^{2K^+}$) detected during pulsing between -1.8 V and various oxidation potentials (1.0, 1.4, and 1.7 V; 200 ms each). Pulses were applied after a 20 s delay to establish a baseline without pulsing. Signal acquisition continued for 1.5 minutes, with pulse effects becoming apparent after ~30 s (20 s initial delay + ~10 s transfer delay). Experimental conditions: 2 mM 2-phenylpropionic acid, 2 mM KPF_6 in 1 M water in acetone, under 0.12 bar O_2 overpressure.

2.4. Mass spectra recorded during optimization of the oxidation pulses

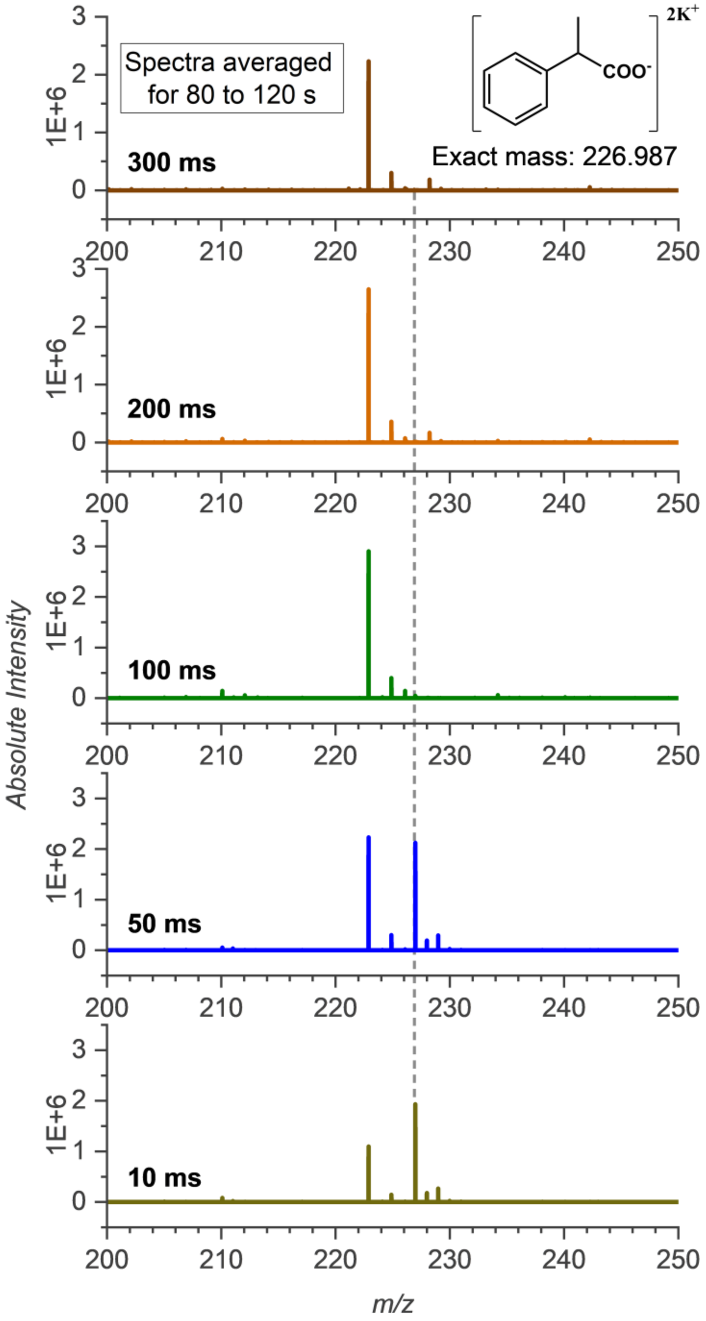

Figure S7: Mass spectra of 2-phenylpropionate ($[RCOO^-]^{2K^+}$) (m/z 226.987) detected during pulsing between -1.8 V (ORR) and 1.7 V (carboxylate oxidation). The reduction pulse (-1.8 V) was fixed at 200 ms, while the oxidation pulse (1.7 V) was varied from 10 to 300 ms (as indicated). Spectra were averaged over 80 - 120 s. Experimental conditions: 2 mM 2-phenylpropionic acid and 2 mM KPF_6 in 1 M water in acetone, under 0.12 bar O_2 over pressure.

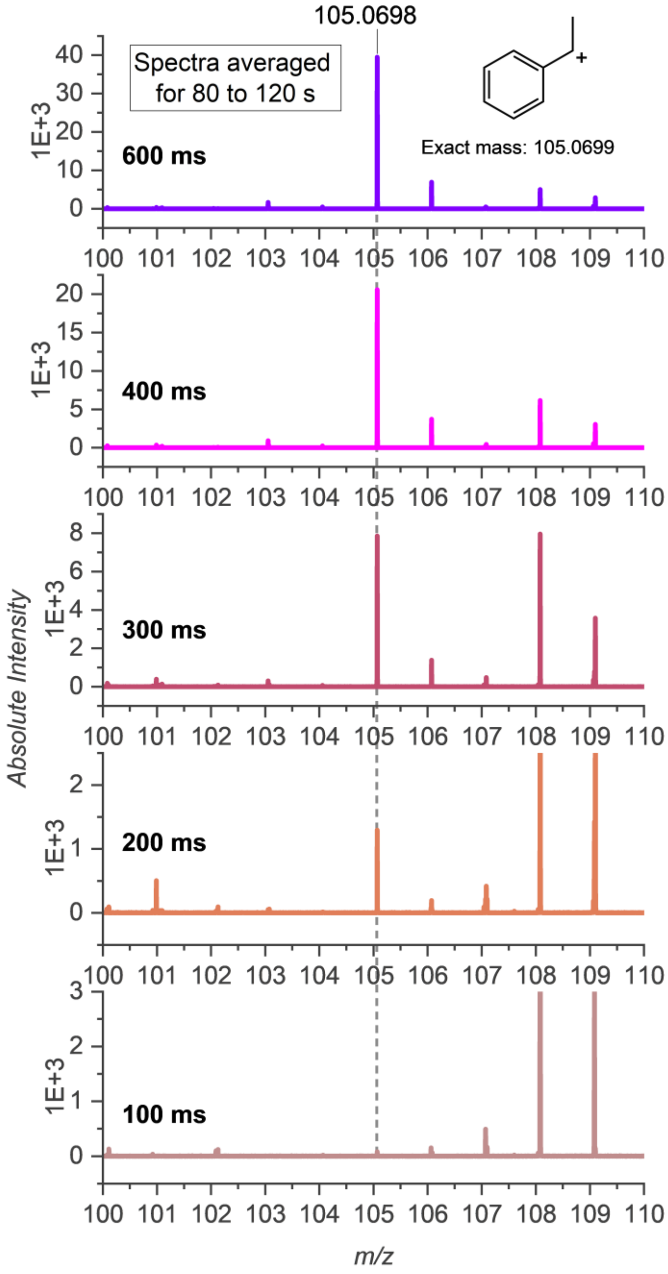

Figure S8: Mass spectra of carbocations (m/z 105.070) detected during pulsing between -1.8 V (ORR) and 1.7 V (carboxylate oxidation). The reduction pulse (-1.8 V) was fixed at 200 ms, while the oxidation pulse (1.7 V) was varied from 100 to 600 ms (as indicated). Spectra were averaged over 80 - 120 s. Experimental conditions: 2 mM 2-phenylpropionic acid and 2 mM KPF_6 in 1 M water in acetone, under 0.12 bar O_2 overpressure.

2.5 ^{1}H NMR after 5 h bulk electrolysis with 200 ms pulses

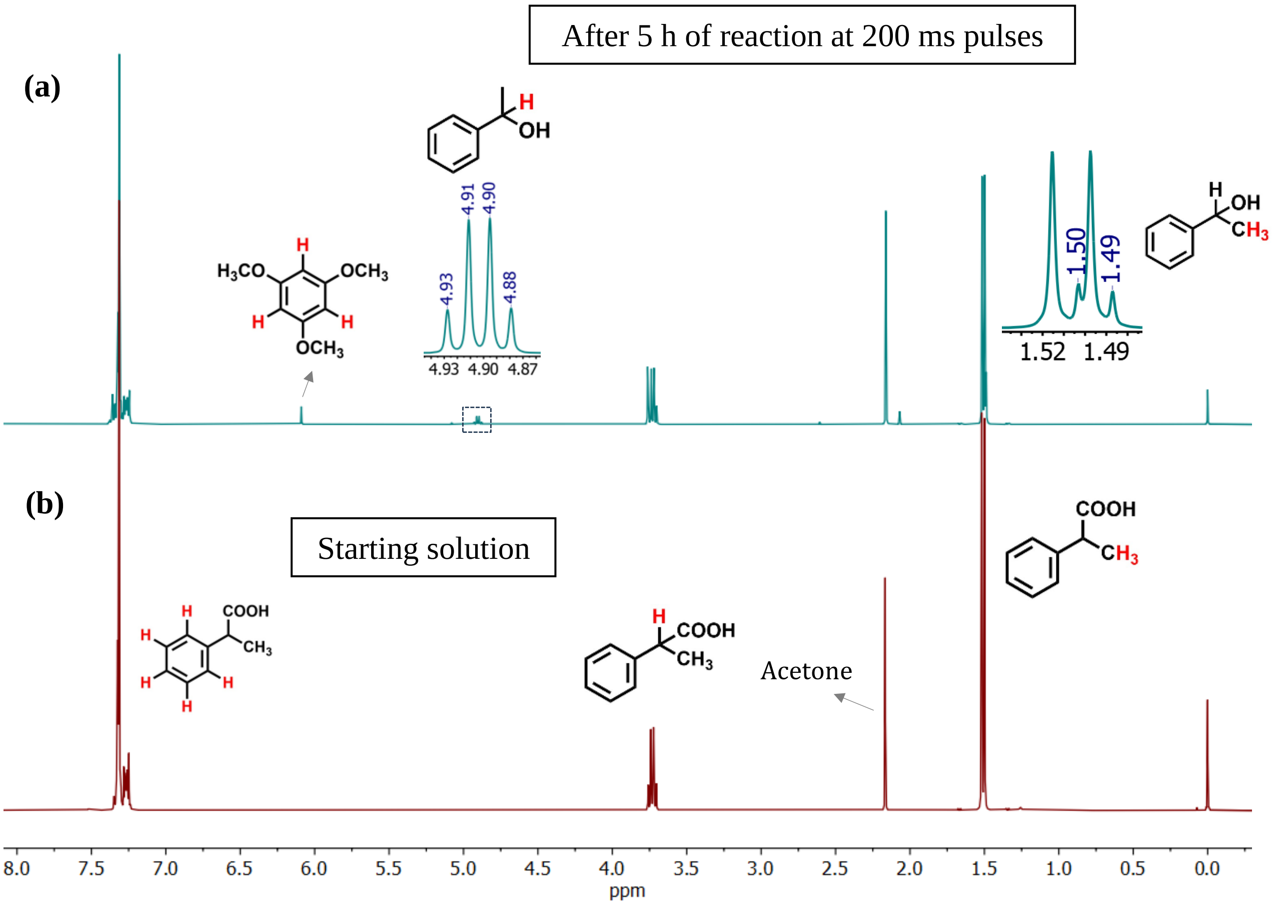

Figure S9: ^{1}H NMR spectra (400 MHz, in $CDCl_3$) of the reaction mixture before (a) and after (b) bulk electrolysis for 5 hours with a polarity switching at 200 ms interval at an applied cell voltage of 3.5 V. Experimental conditions: 2-phenylpropionic acid (125 mM) with KPF_6 electrolyte (100 mM) in 1 M water in acetone (8 mL). Toray carbon paper electrodes of identical dimensions were used, each with a wetted surface area of 2 cm^2. A constant cell voltage of 3.5 V was applied, alternating the electrode polarity every 200 ms. Oxygen was continuously bubbled through the reaction mixture at a head space pressure of 35 mbar, and the solution was stirred at 600 rpm throughout the electrolysis. The NMR yield was calculated using the aromatic-H (3H, s) signal of the internal standard at 6.09 ppm and the benzylic-H of the product 1-phenylethanol (1H, q, 4.93-4.88 ppm).

2.6 ^{1}H NMR after 24 h bulk electrolysis with 300 ms pulses

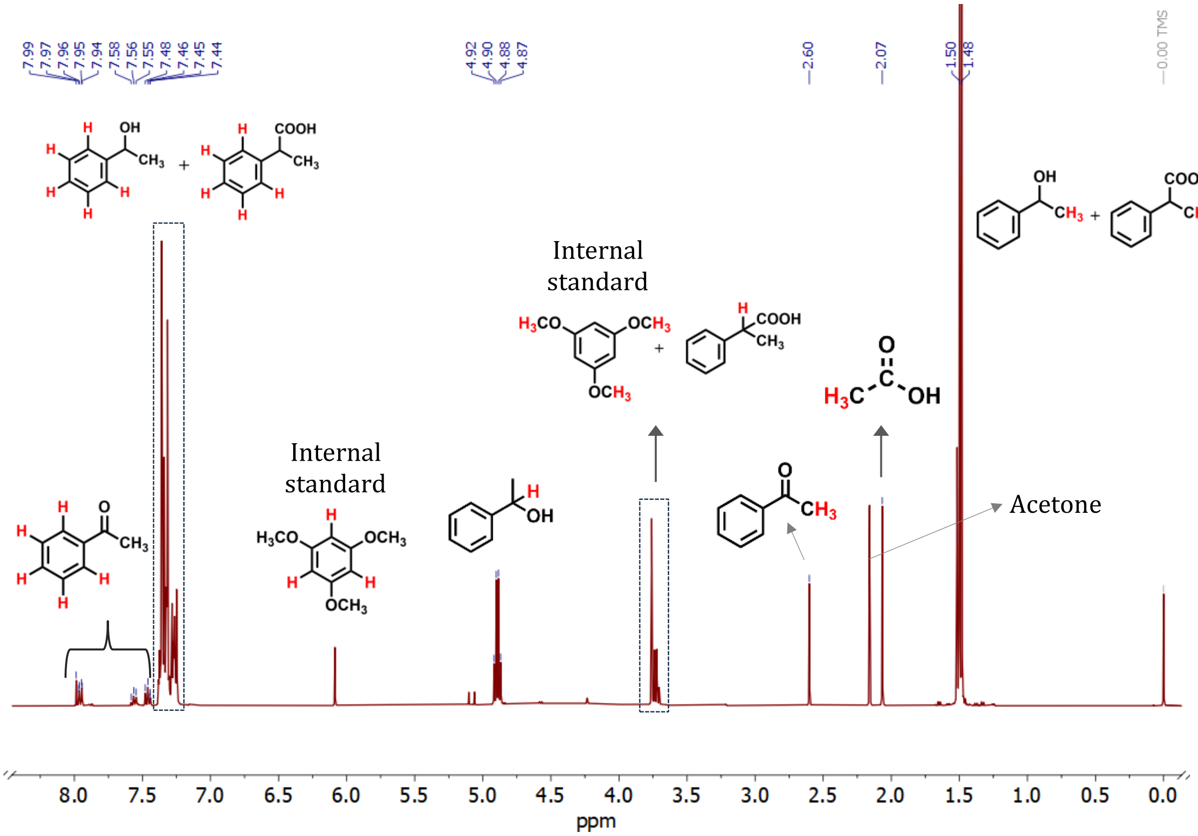

Figure S10: ^{1}H NMR spectrum (400 MHz, in $CDCl_3$) of the reaction mixture after bulk electrolysis for 24 hours with polarity switching at 300 ms intervals at an applied cell voltage of 3.5 V. Experimental conditions: 2-phenylpropionic acid (125 mM) with KPF_6 electrolyte (100 mM) in 1 M water in acetone (8 mL). 1-phenylethanol was the major product (yield ~ 60 %), with acetophenone detected as a minor product (yield ~ 4.3 %) from overoxidation. A singlet at 2.07 ppm corresponds to acetic acid, formed as a side product by the solvent (acetone) oxidation.

1-phenylethanol; ^{1}H NMR (400 MHz, $CDCl_3$) : δ 7.44 - 7.22 (m, 5H), 4.93 – 4.88 (q, 6.5 Hz, 1H), 1.50 - 1.49 (d, 3H).

Acetophenone; ^{1}H NMR (400 MHz, $CDCl_3$): δ 7.99 - 7.94 (m, 2H), 7.58 - 7.55 (m, 1H), 7.48 - 7.44 (m, 2H), 2.60 (s, 3H).

2.7 Electrodes after 24 h of bulk electrolysis at 300 ms pulses

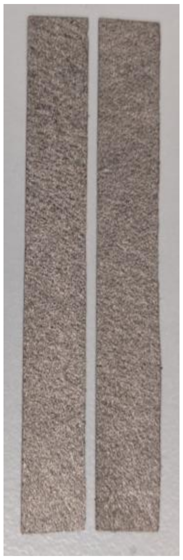

Figure S11: Electrodes showing no sign of electrode fouling or deposition after 24 h of bulk electrolysis with polarity switching at 300 ms intervals at an applied cell voltage of 3.5 V. Experimental conditions: 2-phenylpropionic acid (125 mM) with KPF_6 electrolyte (100 mM) in 1 M water in acetone (8 mL).

2.8 ^{1}H NMR after 5 h bulk electrolysis under DC

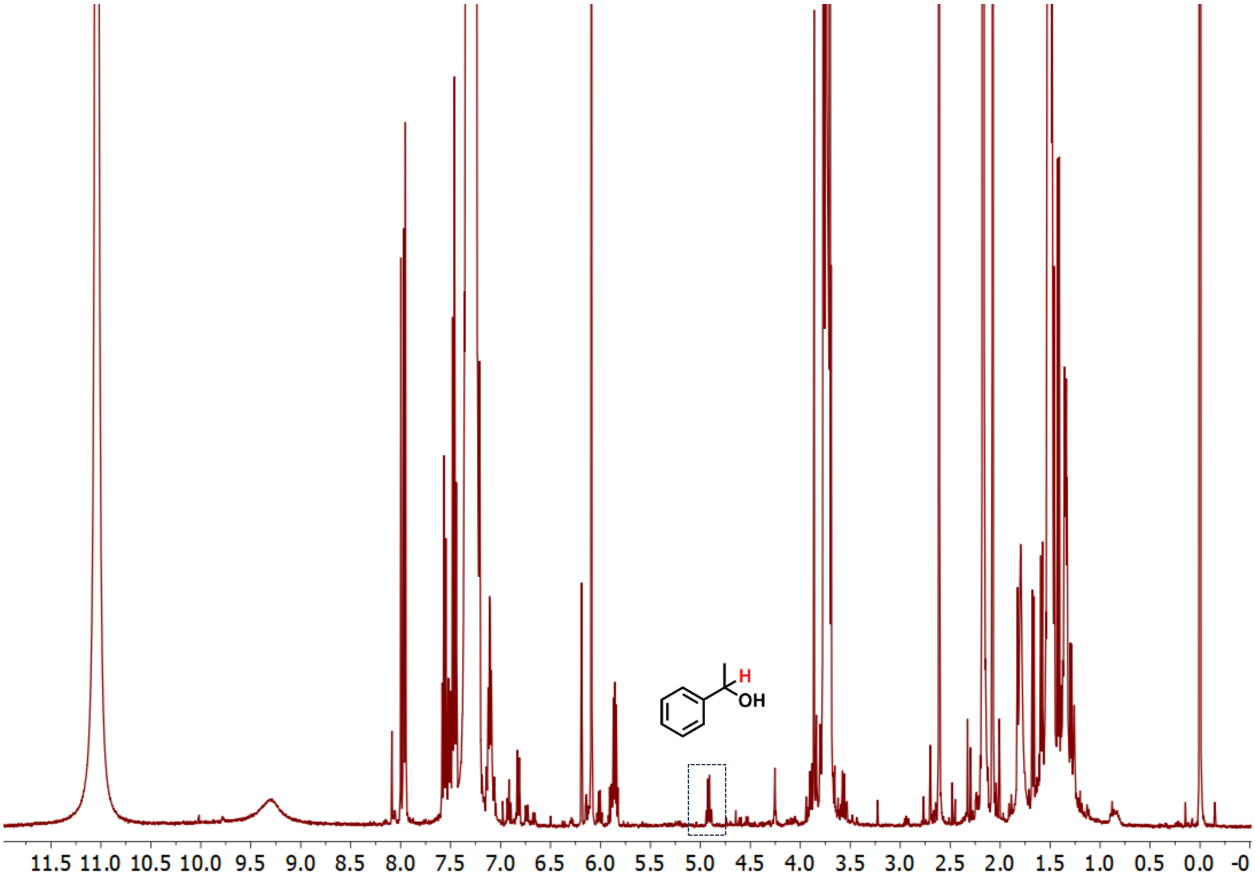

Figure S12: ^{1}H NMR spectrum (400 MHz, in $CDCl_3$) of the reaction mixture after bulk electrolysis for 5 hours at an applied cell voltage of 3.5 V without alternating the polarity (DC). Experimental conditions: 2-phenylpropionic acid (125 mM) with KPF_6 electrolyte (100 mM) in 1 M water in acetone (8 mL). Toray carbon paper electrodes of identical dimensions were used, each with a wetted surface area of 2 cm^2. Oxygen was continuously bubbled through the reaction mixture at a head space pressure of 35 mbar, and the solution was stirred at 600 rpm throughout the electrolysis.

2.9 Electrodes after 5 h of bulk electrolysis under DC

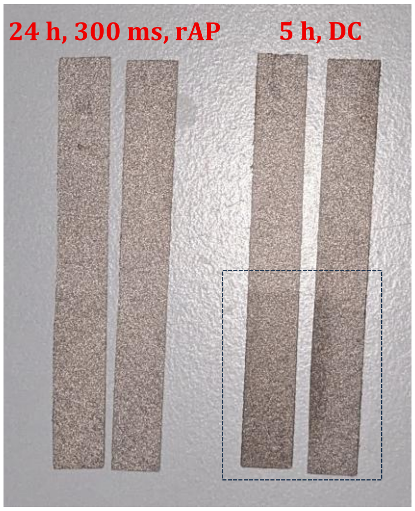

Figure S13: Electrode showing the deposition after 5 h of bulk electrolysis at 3.5 V without alternating the polarity under DC, compared to the electrodes after 24 h at 300 ms with alternating polarity. Experimental conditions: 2-phenylpropionic acid (125 mM) with KPF_6 electrolyte (100 mM) in 1 M water in acetone (8 mL).

2.10 ^{1}H NMR after 5 h AC electrolysis under argon with 1 M H_2O (300 ms pulses)

2.10.1 Bulk electrolysis in acetone; ^{1}H NMR in $CDCl_3$ after solvent removal

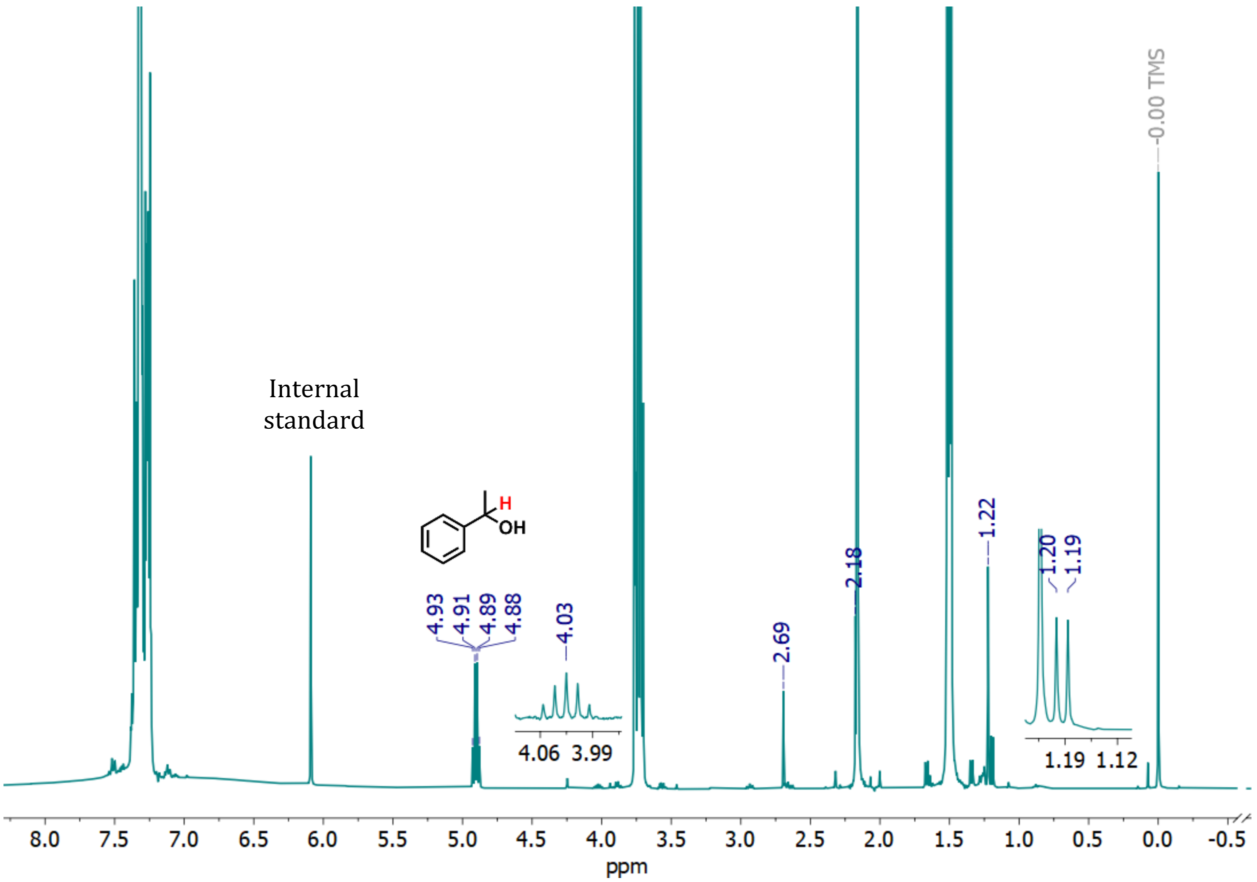

Figure S14: ^{1}H NMR spectrum (400 MHz, in $CDCl_3$) of the reaction mixture after bulk electrolysis for 5 hours in acetone-d_6 with a polarity switching at 300 ms interval at an applied cell voltage of 3.5 V under an Argon atmosphere with 1M water. Experimental conditions: 2-phenylpropionic acid (125 mM) with KPF_6 electrolyte (100 mM) in 1 M water in acetone (8 mL). Toray carbon paper electrodes of identical dimensions were used, each with a wetted surface area of 2 cm^2. Oxygen was removed before the experiment by purging with Argon for 30 min, and the headspace of the cell was under argon pressure during the reaction. The solution was stirred at 600 rpm throughout the electrolysis.

The NMR spectrum shows the product 1-phenylethanol along with additional signals from acetone reduction. A small signal at 4.03 ppm, appearing as a quintet due to low intensity, and a doublet at ~1.2 ppm correspond to isopropanol. Some isopropanol may have been lost during acetone evaporation under vacuum. The peak at 1.22 ppm likely corresponds to 2,3-dimethyl-2,3-butanediol (pinacol), with other signals attributed to related acetone reduction

products (e.g., 3,3-dimethyl-2-butanone formed by pinacol rearrangement). No acetophenone or acetic acid was detected.

To avoid isopropanol loss during evaporation, the experiment was performed in deuterated acetone, and the reaction mixture was directly analyzed in acetone-d_6 without solvent removal.

2.10.2 Bulk electrolysis in acetone-d6 and direct 1H NMR

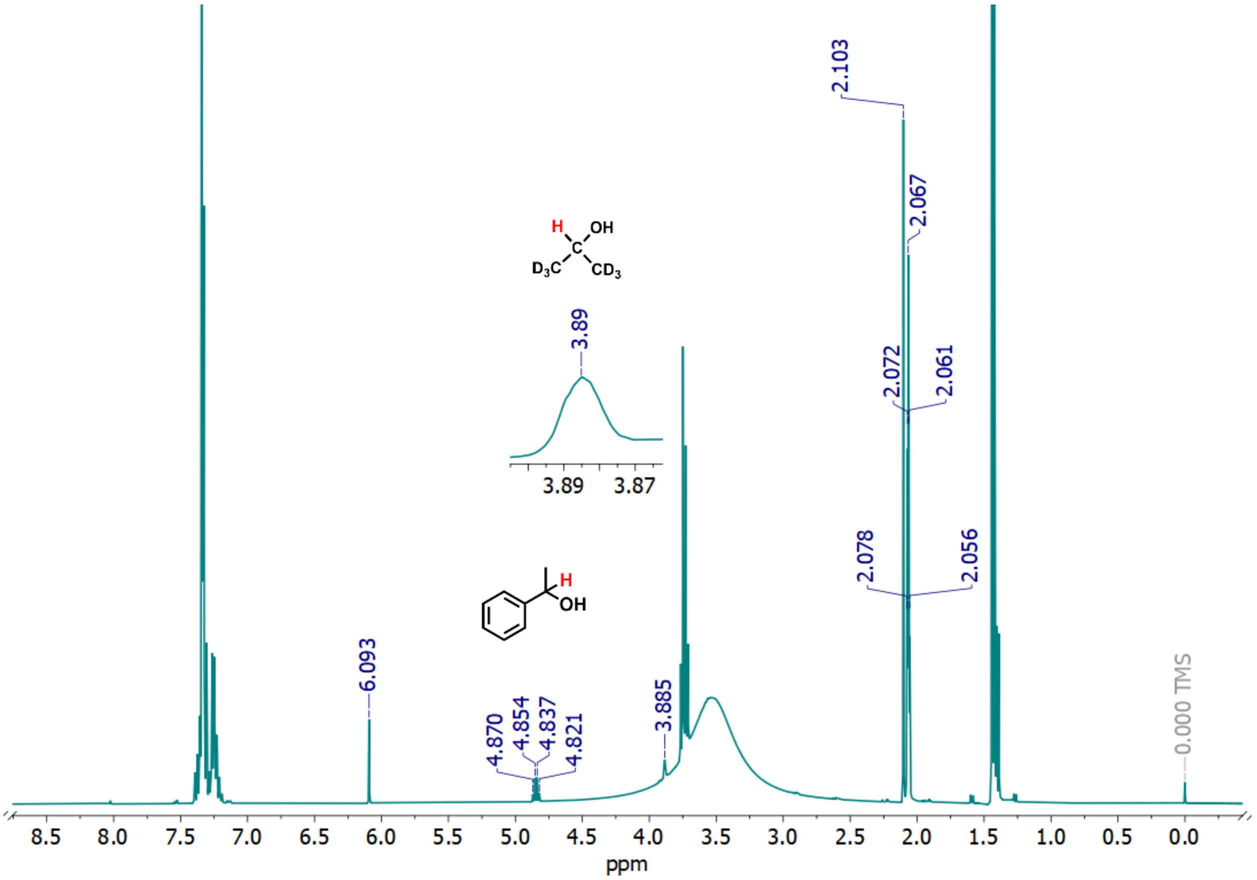

Figure S15: ^{1}H NMR spectrum (400 MHz, in acetone-d$_6$) of the reaction mixture after bulk electrolysis in acetone-d$_6$ for 5 hours with a polarity switching at a 300-ms interval at an applied cell voltage of 3.5 V under an Argon atmosphere with 1M water. Experimental conditions: 2-phenylpropionic acid (125 mM) with KPF_6 electrolyte (100 mM) in 1 M water in acetone-d$_6$ (8 mL). Toray carbon paper electrodes of identical dimensions were used, each with a wetted surface area of 2 cm^2. Oxygen was removed from the experiment by purging with argon for 20 minutes, and the headspace of the cell was maintained under argon pressure during the reaction. The solution was stirred at 600 rpm throughout the electrolysis. The reaction mixture in acetone-d$_6$ was directly analyzed by NMR.

The NMR spectrum shows the product 1-phenylethanol along with a broad water peak and a shoulder at 3.89 ppm. The peak at 3.89 ppm corresponds to the methine proton of isopropanol ($(CD_3)_2CHOH$), appearing broad due to coupling with deuterium. All other acetone reduction signals are now absent in acetone-d$_6$, confirming they originated from reactions with acetone. The signal at 2.07 ppm (quintet) corresponds to acetone-d$_5$, and the singlet at 2.10 ppm to non-deuterated acetone from H/D exchange with water.

2.11 ^{1}H NMR after 5 h AC electrolysis under oxygen without H_2O (300 ms pulses)

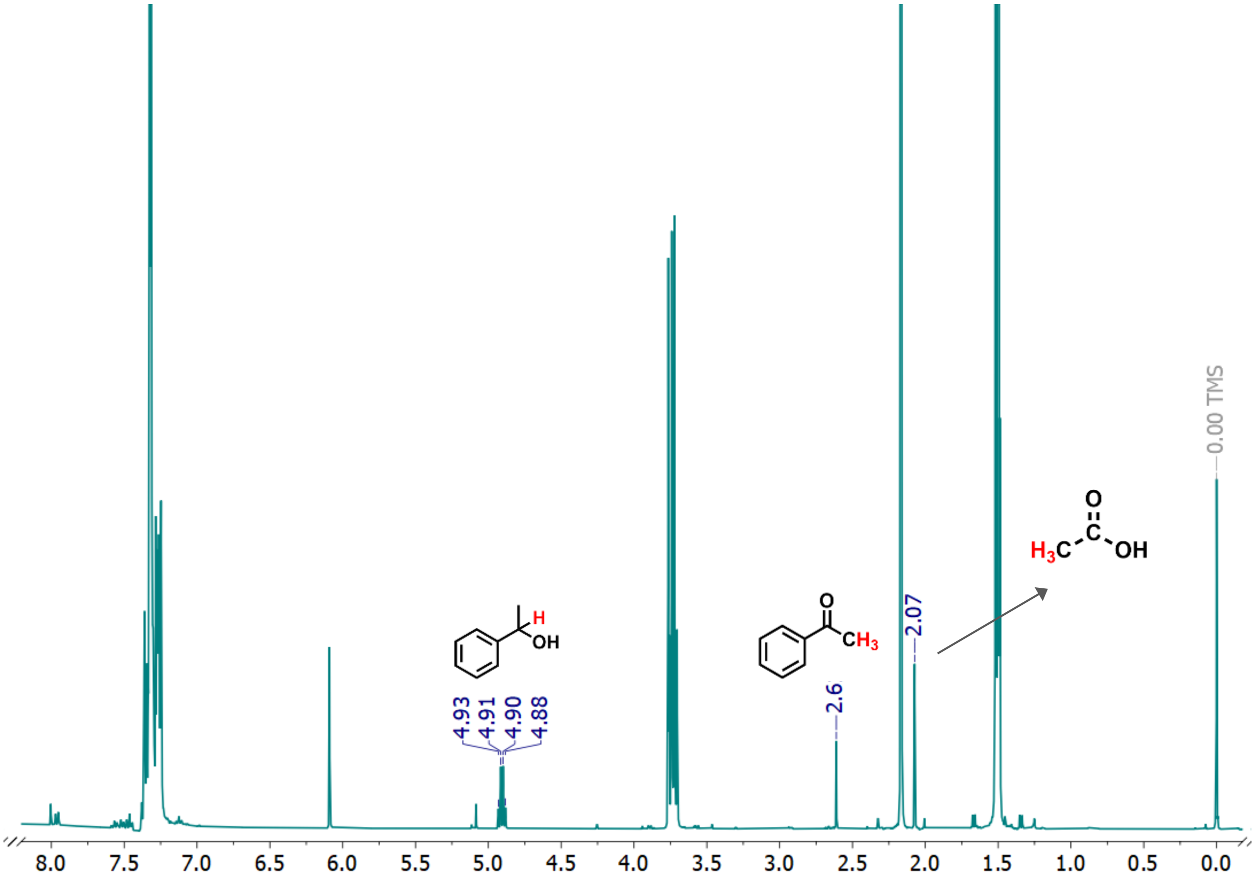

Figure S16: ^{1}H NMR spectrum (400 MHz, in $CDCl_3$) of the reaction mixture after bulk electrolysis for 5 hours under O_2 in the absence of water with a polarity switching at 300 ms interval at an applied cell voltage of 3.5 V. Experimental conditions: 2-phenylpropionic acid (125 mM) with KPF_6 electrolyte (100 mM) in acetone (8 mL). Toray carbon paper electrodes of identical dimensions were used, each with a wetted surface area of 2 cm^2. Oxygen was continuously bubbled through the reaction mixture at a head space pressure of 35 mbar, and the solution was stirred at 600 rpm throughout the electrolysis.

In the presence of oxygen, isopropanol or other acetone reduction products were not observed. Instead, as in the experiments with both O_2 and water, acetophenone and acetic acid were detected.

2.12 Summary of bulk electrolysis

The bulk electrolysis was performed with ElectraSyn under standard conditions: 2-phenylpropionic acid (125 mM) with KPF_6 electrolyte (100 mM) in 1 M water in acetone (8 mL). Toray carbon paper electrodes of identical dimensions were used, each with a wetted surface area of 2 cm^2. A cell voltage of 3.5 V was applied with alternating electrode polarity. Oxygen was continuously bubbled through the reaction mixture at an oxygen overpressure of 35 mbar, and the solution was stirred at 600 rpm throughout 5 hours of electrolysis.

Table S1. Summary of bulk pulsed paired decarboxylative hydroxylation of 2-phenylpropionic acid.

Pulse length [ms]	Charge [F/mol][a]	1-phenyl ethanol	Acetophenone	Alcohol selectivity	Acetic acid Mole ratio (1-phenylethanol : acetic acid)	Isopropanol Mole ratio 1-phenylethanol : isopropanol)
10	11.66	1.05 %	Traces	99.9 %	1 : 0.08	None
50	9.25	7.81 %	0.13 %	98.4 %	1 : 0.16	None
100	7.59	8.94 %	0.15 %	98.3 %	1 : 0.17	None
200	6.99	12.09 %	0.24 %	98.0 %	1 : 0.14	None
300	6.36	13.90 %	0.29 %	97.9 %	1 : 0.11	None
500	7.40	16.20 %	0.43 %	97.4 %	1 : 0.08	None
300[b]	37.25	61.22 %	4.40 %	93.3 %	1 : 0.12	None
DC	2.82	0.82 %	4.59 %	15.3 %	1 : 3.79	None
300[c]	5.28	8.36 %	None*	100 %	None*	1 : 0.44†
300[d]	6.09	9.09 %	0.95 %	90.5 %	1: 0.23	None

a. Charge passed per mol of 2-phenylpropionic acid
b. Standard conditions, but with a reaction time of 24 hours.
c. Without oxygen (under argon), with 1 M water
d. With oxygen, without the addition of water
* Isopropanol and other acetone reduction products observed
† Approximate ratio obtained from the acetone-d6 experiment (after water suppression NMR).

1-Phenylethanol was the major product, with acetophenone formed in minor amounts via overoxidation. Acetic acid was detected as a side product of acetone oxidation. Electrolysis in the absence of oxygen (under argon) with 1 M water produced isopropanol and other acetone reduction products, but no acetophenone or acetic acid. In contrast, all experiments conducted under oxygen showed acetophenone formation and acetone oxidation to acetic acid, with no acetone-reduction products observed, as the ORR occurs preferentially instead. Electrolysis under O_2 without water resulted in lower selectivity, with increased overoxidation of 1-phenylethanol to acetophenone and a reduced 1-phenylethanol: acetic acid ratio. These results suggest that water suppresses the overoxidation of both 1-

phenylethanol and acetone through preferential self-oxidation. At the same time, the reactive oxygen species generated from water oxidation also contribute constructively to the product formation, thereby enhancing both overall yield and selectivity when O_2 and water are present.

3. Experimental Results for 4-phenylbutyric acid

3.1. VESI-MS monitoring – ion traces

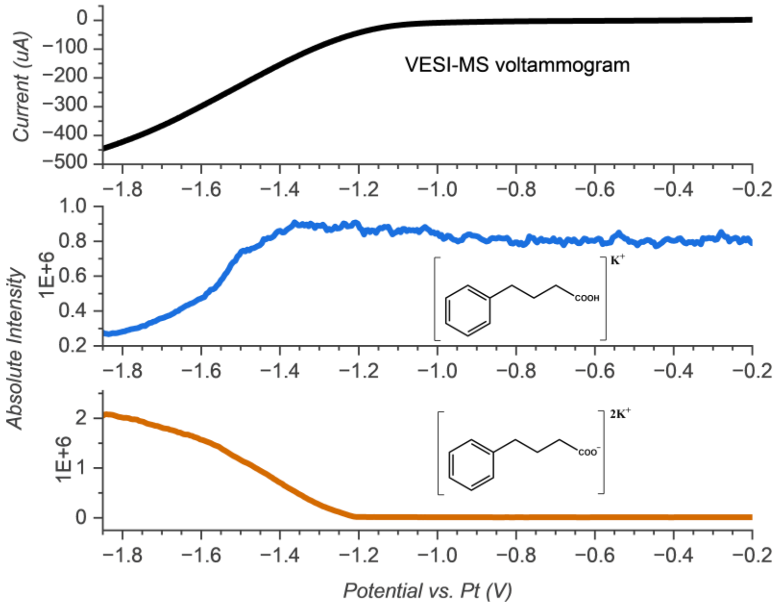

Figure S17: The VESI-MS monitoring of oxygen reduction reaction by 4-phenylbutyric acid (2 mM) and signal of carboxylate ion detected. Experimental conditions: 2 mM 4-phenylbutyric acid, 2 mM KPF_6 in 1 M water in acetone, under 0.12 bar O_2 overpressure.

3.2. VESI-MS monitoring – mass spectra

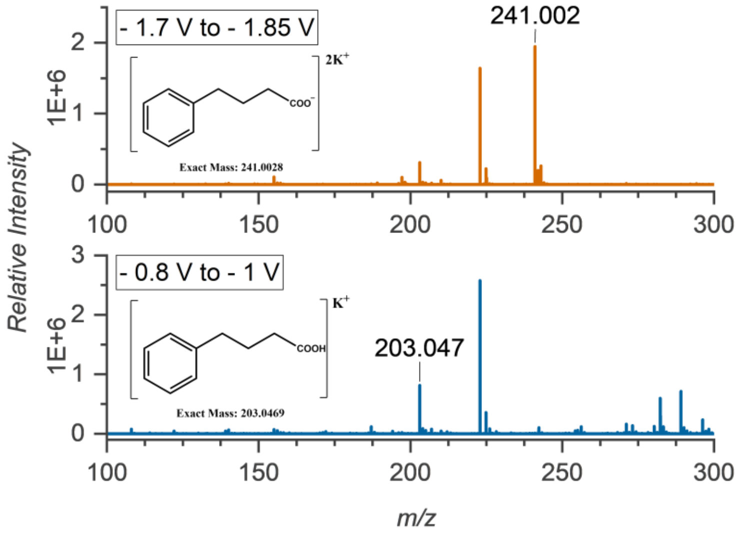

Figure S18: Mass spectra of 4-phenylbutyric acid and its carboxylate ion (m/z 241.002) recorded during the VESI-MS scan toward the oxygen reduction reaction, averaged over the indicated voltage ranges (vs. Pt). Experimental conditions: 2 mM 4-phenylbutyric acid, 2 mM KPF_6 in 1 M water in acetone, under 0.12 bar O_2 overpressure.

3.3. Optimization of the reduction pulse length – traces of carboxylate ion

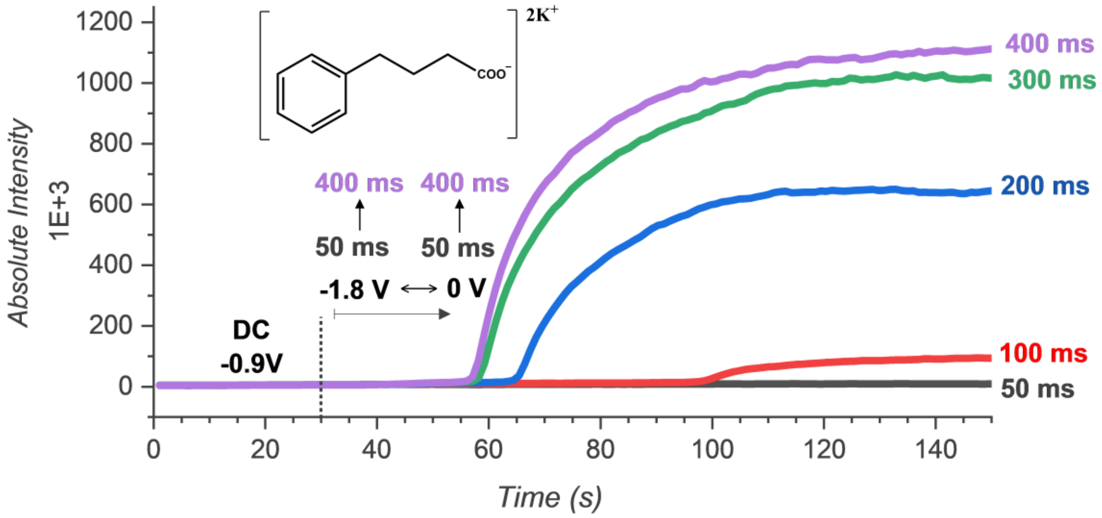

Figure S19: Tuning the reduction pulse length for the maximum carboxylate formation. Extracted ion traces of 4-phenylbutyrate ($[RCOO^{-}]^{2K^{+}}$) detected during pulsing between -1.8 V (ORR) and 0 V (no reaction), with varying pulse lengths. Experimental conditions: 2 mM 4-phenylbutyric acid, 2 mM KPF_6 in 1 M water in acetone, under 0.12 bar O_2 overpressure. Pulses were applied after a 30 s delay to establish a baseline without pulsing.

3.4. Optimization of the reduction pulses – mass spectra of carboxylate

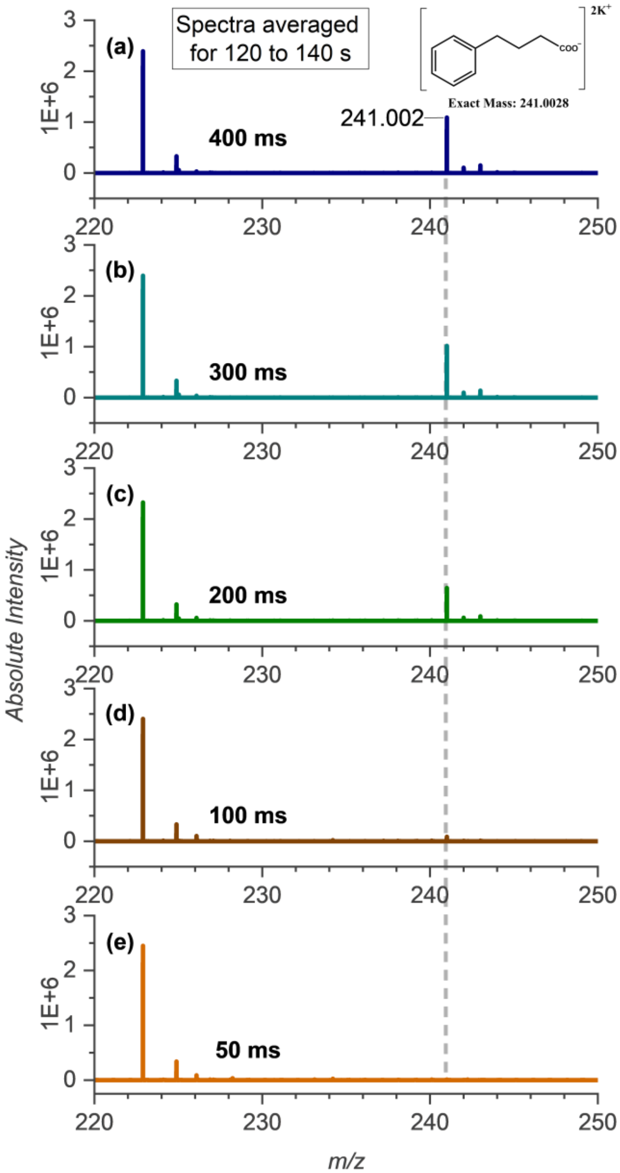

Figure S20: Mass spectra of 4-phenylbutyrate ($[RCOO^-]^{2K^+}$) (m/z 241.002) detected during pulsing between -1.8 V (ORR) and 0 V (no reaction), with varying pulse lengths. Spectra were averaged over 120 - 140 s for: (a) 400 ms, (b) 300 ms, (c) 200 ms, (d) 100 ms, and (e) 50 ms. Experimental conditions: 2 mM 4-phenylbutyric acid, 2 mM KPF_6 in 1 M water in acetone, under 0.12 bar O_2 overpressure.

3.5. Estimation of the oxidation pulse potential

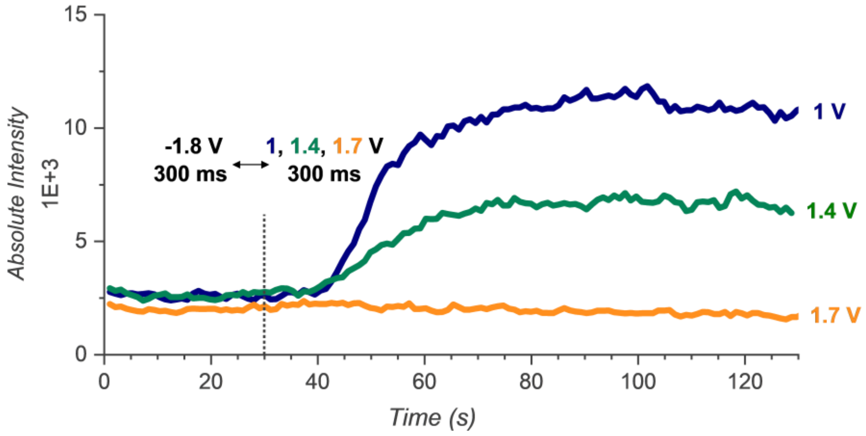

Figure S21: Extracted ion traces of 4-phenylbutyrate ($[RCOO^{-}]^{2K^{+}}$) detected during pulsing between -1.8 V (300 ms) and different oxidation potentials (1.0, 1.4, and 1.7 V; 300 ms each). Pulses were applied after a 30-second delay to establish a baseline without pulsing. Signal acquisition continued for 1.5 minutes, with pulse effects becoming apparent after ~40 s (30 s initial delay + ~10 s transfer delay). Experimental conditions: 2 mM 4-phenylbutyric acid, 2 mM KPF_6 in 1 M water in acetone, under 0.12 bar O_2 overpressure.

3.6. Optimization of the oxidation pulse length – traces of carboxylate ion

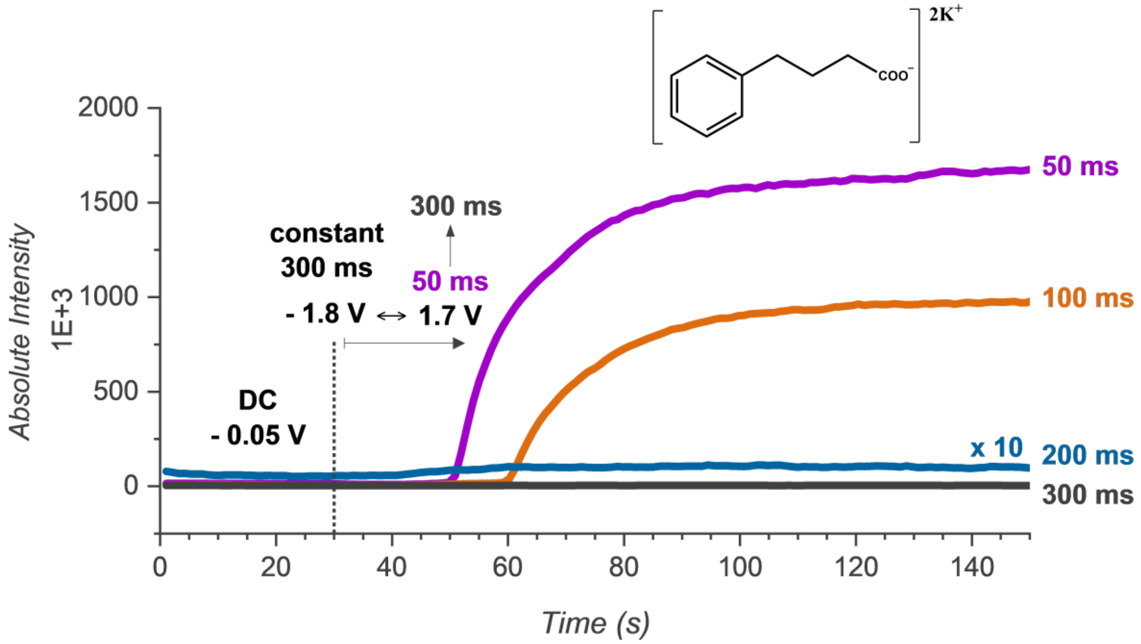

Figure S22: Extracted ion traces of 4-phenylbutyrate ($[RCOO^-]^{2K^+}$) detected during pairing the 300-ms-long reduction pulses (at -1.8 V) with the oxidation pulses (at 1.7 V). With the symmetrical 300-ms-long oxidation pulse, the carboxylate generated from the reduction pulse is almost quantitatively depleted.

3.7. Optimization of the oxidation pulse length – traces of carbocation

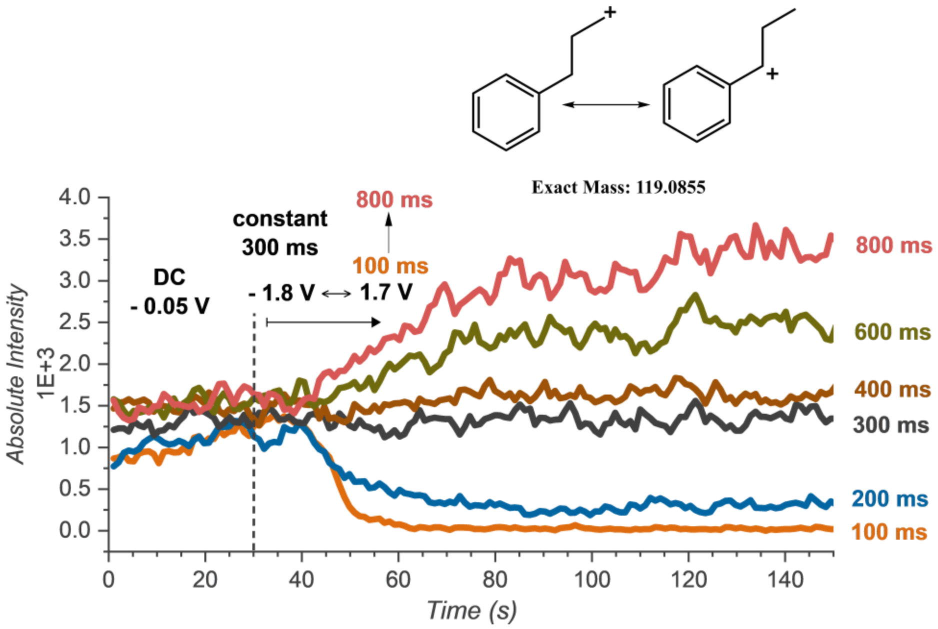

Figure S23: Extracted ion traces of carbocation detected during pairing the 300-ms-long reduction pulses (at -1.8 V) with the oxidation pulses (at 1.7 V). With the symmetrical 300-ms-long oxidation pulse, the carboxylate generated from reduction pulse is almost quantitatively depleted.

3.8. Optimization of the oxidation pulses – mass spectra of carboxylate

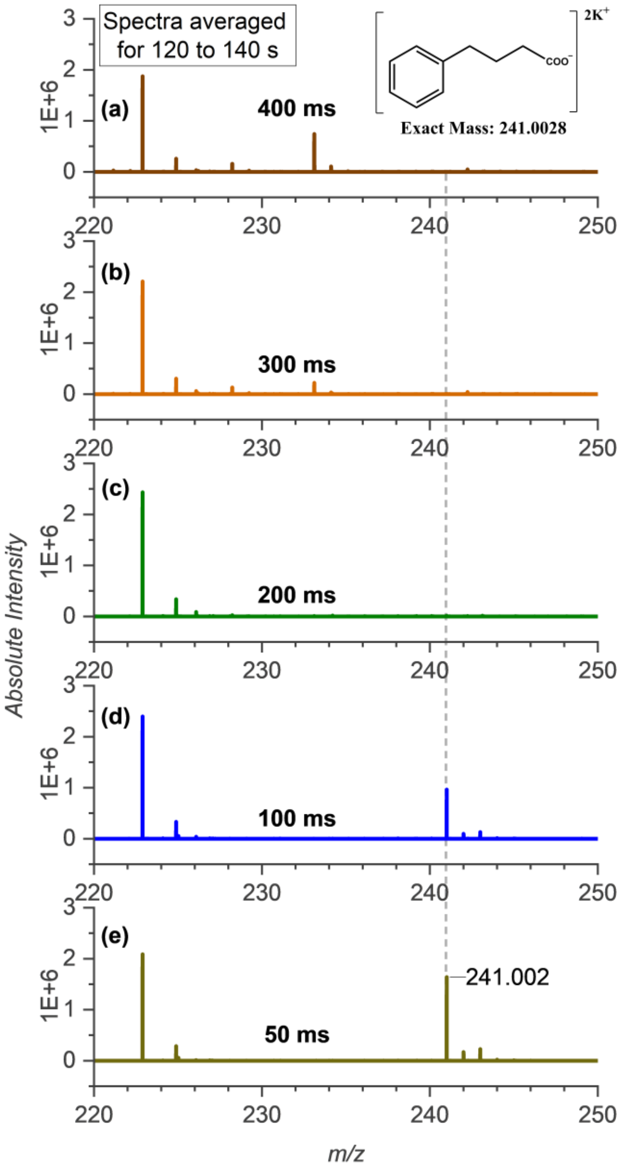

Figure S24: Mass spectra of 4-phenylbutyrate ($[RCOO^-]^{2K^+}$) (m/z 241.002) detected during pulsing between -1.8 V (ORR) and 1.7 V (carboxylate oxidation). The reduction pulse (-1.8 V) was fixed at 300 ms, while the oxidation pulse (1.7 V) was varied from 50 to 400 ms (as indicated). Spectra were averaged over 120 - 140 s. Experimental conditions: 2 mM 4-phenylbutyric acid, 2 mM KPF_6 in 1 M water in acetone, under 0.12 bar O_2 overpressure.

3.9. Optimization of the oxidation pulses – mass spectra of carbocation

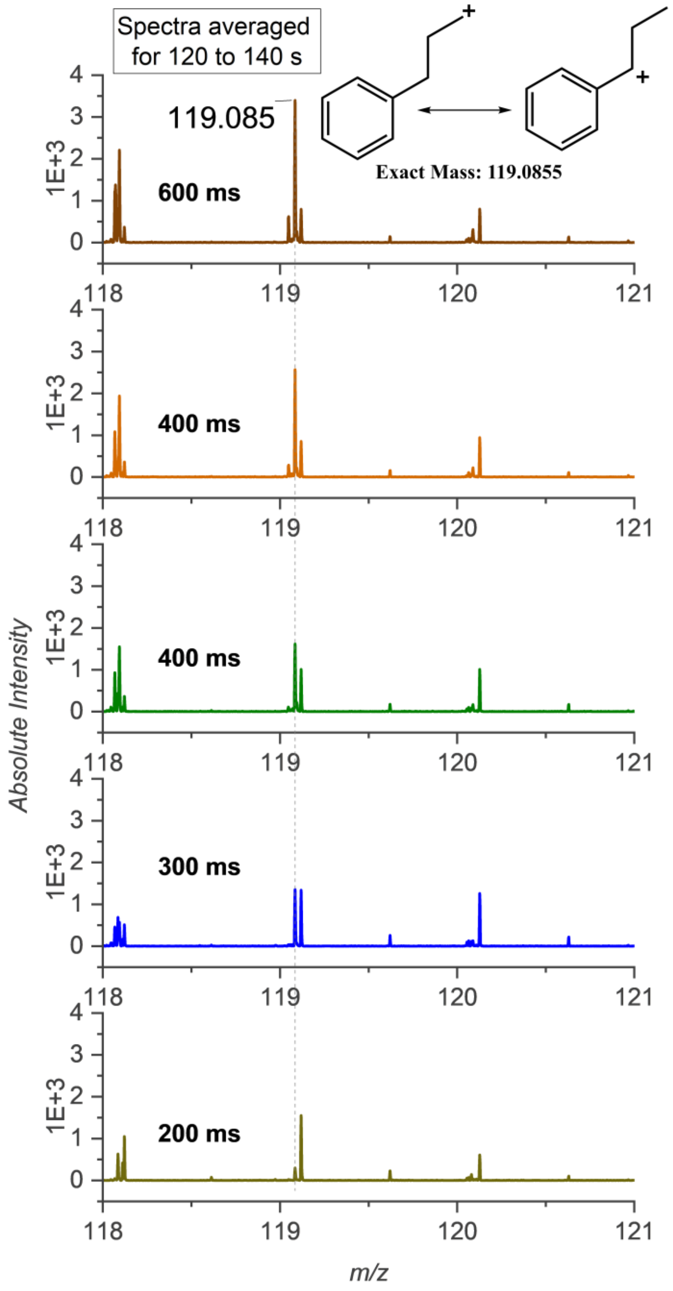

Figure S25: Mass spectra of the carbocation (m/z 119.085) detected during pulsing between -1.8 V (ORR) and 1.7 V (carboxylate oxidation). The reduction pulse (-1.8 V) was fixed at 300 ms, while the oxidation pulse (1.7 V) was varied from 50 to 400 ms (as indicated). Spectra were averaged over 120 - 140 s. Experimental conditions: 2 mM 4-phenylbutyric acid, 2 mM KPF_6 in 1 M water in acetone, under 0.12 bar O_2 overpressure.

3.10. ^{1}H NMR after 5 h bulk electrolysis with 300 ms pulses

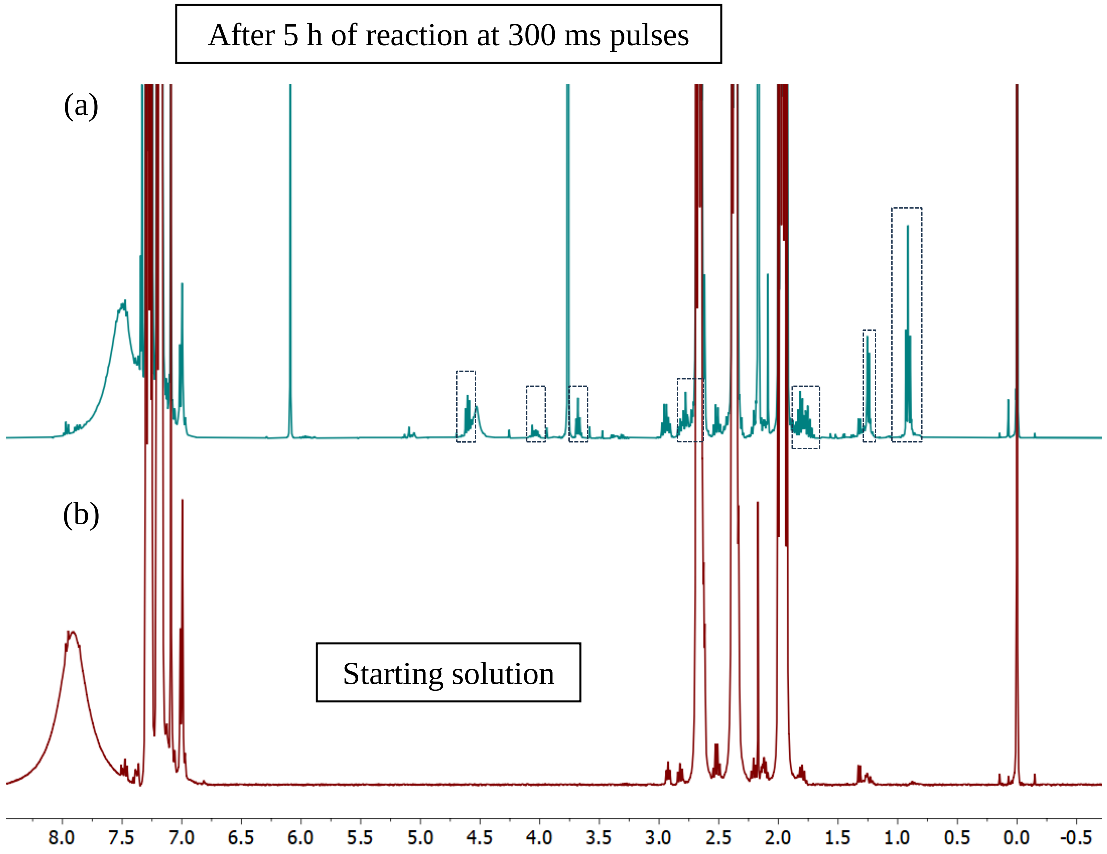

Figure S26: ^{1}H NMR spectra (400 MHz, in $CDCl_3$) of the reaction mixture before (a) and after (b) bulk electrolysis for 5 hours with a polarity switching at 300 ms interval at an applied cell voltage of 3.5 V. Experimental conditions: 4-phenylbutyric acid (125 mM) with KPF_6 electrolyte (100 mM) in 1 M water in acetone (8 mL). Toray carbon paper electrodes of identical dimensions were used, each with a wetted surface area of 2 cm^2. A constant cell voltage of 3.5 V was applied, alternating the electrode polarity every 300 ms. Oxygen was continuously bubbled through the reaction mixture at a headspace pressure of 35 mbar, and the solution was stirred at 600 rpm throughout the electrolysis. The newly formed alcohol peaks are marked.

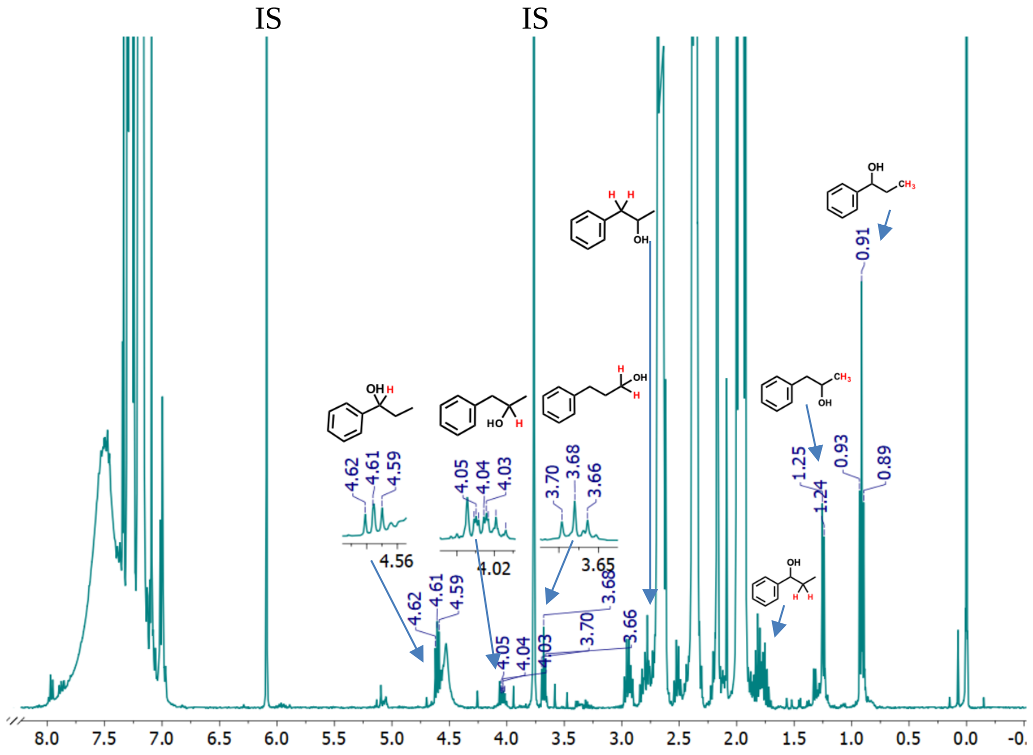

Figure S27: ^{1}H NMR spectra (400 MHz, in $CDCl_3$) of the reaction mixture after bulk electrolysis for 5 hours with a polarity switching at 300 ms interval at an applied cell voltage of 3.5 V. Experimental conditions: 4-phenylbutyric acid (125 mM) with KPF_6 electrolyte (100 mM) in 1 M water in acetone (8 mL). Toray carbon paper electrodes of identical dimensions were used, each with a wetted surface area of 2 cm^2. A constant cell voltage of 3.5 V was applied, alternating the electrode polarity every 300 ms. Oxygen was continuously bubbled through the reaction mixture at a headspace pressure of 35 mbar, and the solution was stirred at 600 rpm throughout the electrolysis.

The proton signal (red) corresponding to the detected alcohols is annotated and highlighted in the spectrum.[4–6] The NMR yield was calculated using the aromatic-H (3H, s) signal of the internal standard at 6.09 ppm and the benzylic-H of the product 1-phenylethanol (1H, q, 4.93-4.88 ppm).

The terminal alkyl radical formed after the carboxylate oxidation can undergo a rearrangement; the yields of the alcohols are: 3-phenyl-1-propanol: ~ 0.52 %, 1-phenyl-2-propanol ~0.85 %, and predominantly more stable 1-phenyl-1-propanol ~1.45 %, with a combined yield of 2.8 %. The selectivity of the alcohol is likely pulse-dependent. A drop in the yield was expected due to the non-benzylic nature of the substrate; further development of the reaction is in progress.

4. References

(1) Koovakattil Surendran, A.; Roithová, J. Decoding Voltammograms at the Molecular Frontier: Integration of Voltammetry and Mass Spectrometry. *Chemistry–Methods* **2024**, *4* (6), e202400003. https://doi.org/10.1002/cmtd.202400003.

(2) Surendran, A. K.; Pereverzev, A. Y.; Roithová, J. Intricacies of Mass Transport during Electrocatalysis: A Journey through Iron Porphyrin-Catalyzed Oxygen Reduction. *J. Am. Chem. Soc.* **2024**, *146* (22), 15619–15626. https://doi.org/10.1021/jacs.4c04989.

(3) Hioki, Y.; Costantini, M.; Griffin, J.; Harper, K. C.; Merini, M. P.; Nissl, B.; Kawamata, Y.; Baran, P. S. Overcoming the Limitations of Kolbe Coupling with Waveform-Controlled Electrosynthesis. *Science (80-.).* **2023**, *380* (6640), 81–87. https://doi.org/10.1126/science.adf4762.

(4) Battilocchio, C.; Deadman, B. J.; Nikbin, N.; Kitching, M. O.; Baxendale, I. R.; Ley, S. V. A Machine-Assisted Flow Synthesis of SR48692: A Probe for the Investigation of Neurotensin Receptor-1. *Chem. – A Eur. J.* **2013**, *19* (24), 7917–7930. https://doi.org/10.1002/chem.201300696.

(5) Kitamoto, Y.; Kuruma, Y.; Suzuki, K.; Hattori, T. Effect of Solvent Polarity on Enantioselectivity in Candida Antarctica Lipase B Catalyzed Kinetic Resolution of Primary and Secondary Alcohols. *J. Org. Chem.* **2015**, *80* (1), 521–527. https://doi.org/10.1021/jo502521e.

(6) Szostak, M.; Spain, M.; Procter, D. J. Electron Transfer Reduction of Carboxylic Acids Using SmI 2 –H 2 O–Et 3 N. *Org. Lett.* **2012**, *14* (3), 840–843. https://doi.org/10.1021/ol203361k.
